# 2022 Update of the Consensus on the Rational Use of Antithrombotics and Thrombolytics in Veterinary Critical Care (CURATIVE) Domain 6: Defining rational use of thrombolytics

**DOI:** 10.1111/vec.13227

**Published:** 2022-07-26

**Authors:** Claire R. Sharp, Marie‐Claude Blais, Corrin J. Boyd, Benjamin M. Brainard, Daniel L. Chan, Armelle de Laforcade, Robert Goggs, Julien Guillaumin, Alex Lynch, Erin Mays, Duana McBride, Tommaso Rosati, Elizabeth A. Rozanski

**Affiliations:** ^1^ School of Veterinary Medicine Murdoch University Murdoch Western Australia Australia; ^2^ Department of Clinical Sciences University of Montreal Saint‐Hyacinthe Quebec Canada; ^3^ Department of Clinical Sciences, Cummings School of Veterinary Medicine Tufts University North Grafton Massachusetts USA; ^4^ Department of Small Animal Medicine and Surgery, College of Veterinary Medicine University of Georgia Athens Georgia USA; ^5^ Department of Clinical Science and Services The Royal Veterinary College London UK; ^6^ Department of Clinical Sciences Cornell University College of Veterinary Medicine Ithaca New York USA; ^7^ Department of Clinical Sciences Colorado State University Fort Collins Colorado USA; ^8^ Department of Clinical Sciences, College of Veterinary Medicine North Carolina State University Raleigh North Carolina USA; ^9^ Veterinary Specialty Services St. Louis Missouri USA; ^10^ VetsNow Manchester Manchester UK; ^11^ Department for Small Animals, Vetsuisse Faculty University of Zurich Zurich Switzerland

**Keywords:** anticoagulant, antiplatelet agent, cats, dogs, thrombosis

## Abstract

**Objectives:**

To systematically review available evidence and establish guidelines related to the use of thrombolytics for the management of small animals with suspected or confirmed thrombosis.

**Design:**

PICO (Population, Intervention, Control, and Outcome) questions were formulated, and worksheets completed as part of a standardized and systematic literature evaluation. The population of interest included dogs and cats (considered separately) and arterial and venous thrombosis. The interventions assessed were the use of thrombolytics, compared to no thrombolytics, with or without anticoagulants or antiplatelet agents. Specific protocols for recombinant tissue plasminogen activator were also evaluated. Outcomes assessed included efficacy and safety. Relevant articles were categorized according to level of evidence, quality, and as to whether they supported, were neutral to, or opposed the PICO questions. Conclusions from the PICO worksheets were used to draft guidelines, which were subsequently refined via Delphi surveys undertaken by the Consensus on the Rational Use of Antithrombotics and Thrombolytics in Veterinary Critical Care (CURATIVE) working group.

**Results:**

Fourteen PICO questions were developed, generating 14 guidelines. The majority of the literature addressing the PICO questions in dogs is experimental studies (level of evidence 3), thus providing insufficient evidence to determine if thrombolysis improves patient‐centered outcomes. In cats, literature was more limited and often neutral to the PICO questions, precluding strong evidence‐based recommendations for thrombolytic use. Rather, for both species, suggestions are made regarding considerations for when thrombolytic drugs may be considered, the combination of thrombolytics with anticoagulant or antiplatelet drugs, and the choice of thrombolytic agent.

**Conclusions:**

Substantial additional research is needed to address the role of thrombolytics for the treatment of arterial and venous thrombosis in dogs and cats. Clinical trials with patient‐centered outcomes will be most valuable for addressing knowledge gaps in the field.

**Abbreviations**
aPTT: activated partial thromboplastin time
ATE: aortic thromboembolism
CRI: constant rate infusion
LAD: left anterior descending
LMWH: low‐molecular‐weight heparin
LOE: level of evidence
LRS: lactated Ringer's solution
PAI‐1: plasminogen activator inhibitor‐1
rscu‐PA: recombinant single‐chain urokinase plasminogen activator
rt‐PA: recombinant tissue plasminogen activator
scu‐PA: single‐chain urokinase plasminogen activator
tPA: tissue plasminogen activator
UFH: unfractionated heparin
uPA: urokinase plasminogen activator
VF: ventricular fibrillation

## 1 INTRODUCTION

Thrombolytic agents (also known as fibrinolytics) are enzymatic plasminogen activators that convert plasminogen to plasmin, which in turn cleaves fibrin to form increasingly smaller protein fragments in the process of fibrinolysis. The endogenous fibrinolytic system is an important component of natural thromboresistance, and includes the natural plasminogen activators tissue plasminogen activator (tPA) and single‐chain urokinase plasminogen activator (scu‐PA; or urokinase plasminogen activator [uPA]).^1^ Plasminogen activator inhibitor (PAI)‐1 is the primary inhibitor of these endogenous fibrinolytic proteins. Pharmacological thrombolytics have been developed to facilitate the lysis of thrombi associated with disease states, with the goal of restoring blood flow through occluded vessels. The predominant pharmacological thrombolytics work by accelerating natural fibrinolysis, although direct thrombolytic enzymes have also been developed.^2^ Three generations of thrombolytics are now used in clinical practice (Table 1).

**TABLE 1:** Select clinical features of established thrombolytic agents in people, classified by generation

Thrombolytic generation	Thrombolytic agent	Half‐life (min)	Fibrin specificity	PAI‐1 inhibition
First generation	Streptokinase	30	–	+++
	Urokinase	15	–	+++
Second generation	Alteplase	4–8	++	+++
Third generation	Reteplase	14–18	+	++
	Tenecteplase	11–20	+++	–

Individual thrombolytics and generations of thrombolytics vary in their half‐life, fibrin specificity, and susceptibility to inhibition by PAI‐1, among other features. Function is directly related to their complex structure, with different components of the molecule conferring specific pharmacokinetic and pharmacodynamic properties.^2^, ^3^ First‐generation thrombolytics (eg, streptokinase, urokinase) are naturally occurring compounds that have negligible fibrin specificity and are significantly inhibited by PAI‐1. The lack of fibrin specificity increases the risk of hemorrhage associated with their use.^1^ Fibrin specificity is improved with second‐generation agents, specifically recombinant tissue plasminogen activator (rt‐PA). Alteplase is the most widely available recombinant single‐chain tPA. Third‐generation products, such as reteplase and tenecteplase, were developed to reduce PAI‐1 inhibition and increase half‐life while maintaining or improving fibrin specificity.^4^ Second‐ and third‐generation thrombolytics are the products of recombinant DNA technology and/or chemical modification that permit molecular optimization of clinical efficacy.^2^

The acute risk of adverse effects with use of thrombolytic agents, notably hemorrhage and reperfusion injury, is significantly higher than with the use of antithrombotics, as outlined in the 2019 Consensus on the Rational Use of Antithrombotics in Veterinary Critical Care (CURATIVE) guidelines.^5^ When considering the use of thrombolytic agents in practice the premise of “*primum non nocere*” (first do no harm) should be considered.

In contrast to veterinary medicine, the use of thrombolytics in human medicine is well established, in part due to the higher risk of acute life‐threatening arterial thrombosis associated with atherosclerosis, myocardial infarction, and stroke. Research over decades, including large well‐designed multicenter randomized clinical trials and metanalyses, has refined indications for thrombolysis to 5 scenarios: (i) ST‐elevation myocardial infarction when cardiac catheterization for revascularization is unavailable, (ii) acute ischemic stroke within 3–4.5 hours of symptom onset, (iii) pulmonary embolism with persistent hypotension, (iv) acute limb ischemia when catheter‐directed thrombolytic agent delivery is feasible, and (v) restoration of flow in occluded central venous catheters.^6^ General contraindications for pharmacological thrombolysis in human medicine are also well established and include (but are not limited to) active internal bleeding, thrombocytopenia (platelet count < 100 × 10^3^/μL), prolonged clotting times, recent anticoagulant use, stroke or traumatic brain injury within 2–3 months, previous intracranial hemorrhage, an intracranial tumor, arteriovenous malformation or aneurysm, recent intracranial or spinal surgery, recent vascular puncture at noncompressible site, and uncontrolled systemic hypertension.

The aim of Domain 6 was to systematically review available evidence and establish guidelines related to the use of thrombolytics for the management of small animals with suspected or confirmed thrombosis.

## 2 MATERIALS AND METHODS

The first CURATIVE guidelines were first published in 2019.^5^ Domain 6 originated from feedback received during the initial CURATIVE consultation process that highlighted the need for guidelines regarding the use of thrombolytics. As previously described, the process of developing consensus guidelines involved formulation of PICO (Population or Patient, Intervention, Control or Comparison, and Outcome) questions, completion of PICO worksheets, development of draft guidelines, and their subsequent refinement via Delphi surveys undertaken by the CURATIVE working group.

Fourteen PICO questions were developed for this domain. The PICO questions were assigned to individual worksheet authors with expertise in the field with a second person assigned to review each PICO question prior to review by the Domain chair. Online literature searches of the Medline and CAB databases were performed as for the other CURATIVE Domains.^5^ Inclusion criteria included pharmacologic thrombolysis of intravascular thrombi in vivo in dogs and cats. Studies were excluded if they only described in vitro experiments, or exclusively addressed ultrasound‐assisted thrombolysis (sonothrombolysis), mechanical or surgical thrombectomy, or the treatment of intravesicular thrombi (eg, urinary bladder). Studies in languages other than English were included when a translation of the work into English was available. Additionally, studies were excluded if they reported the use of thrombolytics that are not commercially available (eg, alfimeprase, YM866).^7^

For Domain 6, the population of interest included dogs and cats (considered separately) and arterial and venous thrombosis. The interventions assessed were the use of thrombolytics, compared to no thrombolytics, with or without anticoagulants or antiplatelet agents. Specific protocols for rt‐PA were also evaluated. Outcomes assessed included efficacy and safety. Regarding efficacy, since endpoints vary among studies, outcomes were broadly considered as being patient‐centered (eg, survival to hospital discharge, return of function) or not patient‐centered (eg, revascularization documented via angiography). Hemorrhage and complications of reperfusion were the specific safety outcomes of interest.

Relevant studies were assessed to determine the level of evidence (LOE) from 1 to 6, methodological quality, relevance to the PICO question, and magnitude of the observed effect supporting or opposing the PICO question. Randomized, controlled, clinical studies in companion animals were considered LOE 1. Controlled clinical studies in companion animals without randomization were considered LOE 2. Laboratory animal studies in dogs or cats were considered LOE 3, with further delineation based on the inclusion of randomization and controls (good quality), lack of randomized controls (fair quality), and studies without controls (poor quality). Retrospective clinical studies using controls but without randomization were considered LOE 4, while case series were LOE 5. Given differences between people and small animals regarding the fibrinolytic system, causes of thrombosis, clinical presentation, diagnostic capabilities, and hospital resources, the consensus decision of the CURATIVE Steering Committee was to exclude LOE 6 studies (human clinical studies). Note that quality assessments of the same study may vary by PICO question, since relevance to the PICO question is a key component of quality. Consistent with previous domains of CURATIVE, guidelines are written as “We recommend” where strong supportive evidence exists, and “We suggest” where the evidence is weak.^5^ Preliminary guidelines were presented at the International Veterinary Emergency and Critical Care Symposium in September 2021. Guidelines (Appendix A) and the proportion of the working group members reaching consensus via Delphi surveys, including the survey round in which consensus was reached, are reported.

## 3 RESULTS

### 3.1 PICO question: Thrombolysis in arterial thrombosis (dogs)

In dogs with suspected or confirmed arterial thrombosis (P), does use of a thrombolytic agent (I) compared to no thrombolytic agent (C) improve any outcomes (O)?

#### 3.1.1 Guidelines

##### Thrombolysis in arterial thrombosis (dogs)

Delphi consensus reached with 15/16, Round 2

- In dogs with confirmed acute arterial thrombosis, particularly where the agent can be delivered within 1 hour of onset of thrombosis, we suggest catheter‐directed intraarterial administration of a thrombolytic agent.
- There is insufficient evidence to determine if thrombolysis improves patient‐centered outcomes.
- No evidence‐based recommendations can be made regarding the use of thrombolytic agents for treatment of chronic arterial thrombosis in dogs.

#### 3.1.2 Evidence summary

Five LOE 3 good‐quality studies supported the PICO question.^8^, ^9^, ^10^, ^11^, ^12^ These studies varied in the mechanism of arterial thrombosis and the specific thrombolytic. Leach and colleagues investigated the use of different streptokinase‐based thrombolytics in an autologous clot injection model of canine coronary artery thrombosis.^8^ In this study, thrombi were allowed to “age” for 45 minutes before study drug administration. Two groups of dogs (*n* = 27 dogs) received no thrombolytic treatment, and none experienced spontaneous reperfusion during the 4‐hour observation period. In contrast, most dogs (34/39 dogs) receiving the intravenously administered streptokinase formulations experienced reperfusion. Additionally, infusion of any streptokinase formulation resulted in substantial decreases in myocardial infarct size. Dogs treated with streptokinase experienced more bleeding from surgical sites (ie, site of femoral vein exposure, thoracotomy, and thrombin injection site in the coronary artery) than saline‐treated dogs.^12^

Rebello and colleagues also reported thrombolytic efficacy using an electrolytic injury canine coronary artery thrombosis model.^9^ This study included various treatment groups, but the most relevant to the PICO question was the comparison between saline control and rt‐PA. All interventions occurred after 30 minutes of thrombotic occlusion. IV rt‐PA therapy resulted in a higher frequency of reperfusion (11/12; 92%) than in saline‐treated control dogs (0/11; 0%). However, 2 dogs in the rt‐PA group experienced ventricular fibrillation (VF) during reperfusion and subsequently died.^9^ Additionally, 7 of 9 surviving dogs had reocclusion, leading to the authors’ recommendation that antiplatelet and anticoagulant therapies be used to maintain coronary blood flow following reperfusion.^9^

Feuerstein and colleagues investigated both anistreplase and tPA in an induced canine coronary artery thrombosis model.^10^ Treatments were administered after 30 minutes of clot aging and the dogs observed for a further 150 minutes. In their first study, a saline control (*n* = 12) was compared to groups of dogs receiving 3 different doses of IV anistreplase (*n* = 12/group). Reperfusion occurred in 0 of 12 control dogs, 1 of 12 (8.3%) in the low‐dose anistreplase group, 5 of 12 (42%) in the medium dose group, and 12 of 12 (100%) in the high‐dose group, with no reocclusion during the observation period. Similarly, the 2 highest dose groups demonstrated decreased time to reperfusion and significant reductions in clot weight postmortem. The second part of their study compared the administration of lactated Ringer's solution (LRS) to 4 doses of tPA administered IV over 60 minutes. No dogs in the LRS group (0/8) or low‐dose tPA group (0/8; 6 μg/kg total dose) reperfused, compared to 3 of 8 (37.5%) in the 30 μg/kg dose group, 6 of 8 (75%) in the 120 μg/kg dose group, and 8 of 8 (100%) in the 480 μg/kg dose group. No dogs experienced reocclusion. Additionally, the 2 highest dose tPA groups demonstrated decreased time to reperfusion and reduced thrombus weight.^10^

Two publications by Badylak and colleagues reported the use of thrombolytics in a dog model of femoral arterial thrombosis.^11^, ^12^ Treatments were commenced after 30 minutes of thrombus aging and thrombolysis was monitored by the decrease in gamma emissions from radiolabeled thrombi. In the first study, dogs receiving intraarterial pro‐urokinase showed greater thrombolysis at 90 minutes (41%–66% lysis) than the control group (15% lysis). In the second study, all groups that received intraarterial urokinase had greater thrombolysis than the control groups. No dogs in these studies had evidence of hemorrhage.^11^, ^12^

Thirteen LOE 3 studies of fair quality supported the PICO question.^13^, ^14^, ^15^, ^16^, ^17^, ^18^, ^19^, ^20^, ^21^, ^22^, ^23^, ^24^, ^25^ These studies included no thrombolytic control groups without randomization of group allocation. Zhang and colleagues injected a fibrin‐rich embolus into the left anterior descending (LAD) coronary artery causing occlusion.^13^ After 60 minutes of LAD occlusion, dogs received unfractionated heparin (UFH) IV, followed by either saline or rt‐PA administered as a loading dose of 0.4 mg/kg, followed by continuous infusion of 1.2 mg/kg over 30 minutes. After 30 minutes, 0.8 mg/kg was infused over the subsequent 60 minutes. When monitored by serial angiography, 5 of 6 dogs in the rt‐PA group had recanalization after 2 hours, compared to 0 of 6 dogs in the control group, resulting in a decrease in infarct size by >50% with no evidence of bleeding.^13^

In a femoral artery thrombosis model, Fu and colleagues demonstrated improved outcomes with intraarterial infusion of miniplasmin or rt‐PA compared to control when infused 30 minutes after thrombus formation.^14^ Rates of reperfusion were 0/6 in the control group, 4/6 in the low‐dose miniplasmin group (with 1/4 reoccluding), 6/6 in the mid‐dose miniplasmin group (0 reocclusion), 6/6 in the high dose miniplasmin group (0 reocclusion), and 5/6 in the rt‐PA group (1/5 reocclusion). Additionally, thrombus mass was lower in all groups receiving a thrombolytic. Hemorrhagic complications were observed in all dogs in the rt‐PA group, but none of the miniplasmin‐treated dogs.^14^

Burke and colleagues compared intraarterial versus IV use of recombinant pro‐urokinase in a femoral arterial thrombosis model.^15^ Clot formation and aging time totaled 45 minutes before thrombolytic drug administration. When administered IV, 6 of 6 dogs receiving pro‐urokinase experienced recanalization, compared to 0 of 5 vehicle‐treated dogs. Similarly, when administered by catheter‐directed intraarterial injection, 5 of 6 dogs receiving pro‐urokinase had recanalization, compared to 1 of 5 vehicle‐treated dogs. Notably, although both IV and intraarterial routes were effective, the IV dose was approximately 100 times greater than that administered via intraarterial injection. Complications of thrombolysis were not reported.^15^

In another model of femoral artery thrombosis, dose‐dependent thrombolysis was seen in dogs treated with IV tPA 60 minutes after thrombus formation.^18^ In a similar model, Suzuki and colleagues^20^ demonstrated that medium‐ and high‐dose rt‐PA constant rate infusion (CRI) (0.2 and 0.4 mg/kg IV over 30 min), but not a low‐dose CRI or bolus doses, resulted in significantly higher thrombolytic rates than the placebo group.^20^

Maki and colleagues compared catheter‐directed intraarterial (ie, intracoronary) administration of urokinase (*n* = 6), or alteplase (*n* = 8), with saline (*n* = 5) in an endothelial injury model of coronary artery thrombosis.^16^ Treatments were administered 30 minutes after thrombus formation with angioscopy used to assess thrombolytic efficacy at 60 minutes. All dogs in the alteplase group had complete thrombolysis, compared to zero dogs in the control group, while urokinase treatment resulted in incomplete lysis with residual thrombi in all animals.^16^

Haberstroh and colleagues evaluated numerous thrombolytic agents in an autologous clot injection model of renal artery thrombosis.^17^ Local and systemic application of thrombolytic agents resulted in complete recanalization, whereas the clot remained stable in control‐treated animals with or without systemic heparinization. Nonetheless, kidney injury, as demonstrated by increased serum urea or creatinine and decreased glomerular filtration rate, remained during 8 weeks follow‐up, despite successful thrombolysis.^17^

Tomaru and colleagues reported the use of thrombolytics in a model of bilateral iliac artery thrombosis where contralateral arteries acted as controls.^19^ In 5 dogs, saline placebo was catheter‐delivered into 1 thrombosed iliac artery, while low‐dose tPA (tisokinase, 50,000 IU) was catheter‐delivered into the other thrombosed artery. After 60 minutes, thrombotic stenosis decreased more in tPA‐treated arteries compared to saline‐treated controls.^19^

Rote and colleagues published 2 studies that supported the PICO question using bilateral carotid artery thrombosis models, where contralateral carotid arteries acted as controls.^21^, ^22^ Local administration of anisoylated plasminogen streptokinase activator complex proximal to the occlusive thrombus restored blood flow, whereas flow was not restored in control arteries. A 70% reocclusion rate was reported, however.^21^

Lu and colleagues reported the efficacy of rt‐PA‐induced thrombolysis with a platelet inhibitor in a combined model of arterial and venous thrombosis in dogs.^23^ The arterial thrombosis component of this model is most relevant to the PICO question. Dogs in the nonthrombolytic control groups experienced sustained occlusive thrombosis, while dogs receiving IV bolus dose thrombolytic (either recombinant single‐chain urokinase plasminogen activator [rscu‐PA] or rt‐PA) demonstrated dose‐dependent thrombolysis. Reocclusion was reduced at higher doses.^23^

Hiro and colleagues investigated rt‐PA‐induced thrombolysis in an autologous clot injection model of coronary artery thrombosis,^24^ where rt‐PA or vehicle controls were administered 30 minutes after thrombus formation. Five dogs in the rt‐PA group received a 30‐minute IV infusion of rt‐PA (10 μg/kg/min), and all experienced thrombolysis. In contrast, no dogs in the vehicle control group experienced thrombolysis. One of 6 dogs in the vehicle‐treated group died of VF, while 2 dogs allocated to the rt‐PA group died of VF during the occlusion or reperfusion.^24^ Gu and colleagues also used rt‐PA (intravenously or intraarterially) to induce thrombolysis in a coronary artery thrombosis model.^25^ Thrombolysis occurred in dogs that received rt‐PA, but not in control‐treated dogs.^25^

A large number of publications report the use of thrombolytics in experimental arterial thrombosis models in dogs (LOE 3), but lack suitable controls thereby precluding their use to address the PICO question.^26^, ^27^, ^28^, ^29^, ^30^, ^31^, ^32^, ^33^, ^34^, ^35^, ^36^, ^37^, ^38^, ^39^, ^40^, ^41^, ^42^, ^43^, ^44^, ^45^, ^46^, ^47^, ^48^, ^49^, ^50^, ^51^, ^52^, ^53^, ^54^, ^55^, ^56^, ^57^, ^58^, ^59^, ^60^, ^61^, ^62^, ^63^, ^64^, ^65^, ^66^, ^67^, ^68^, ^69^, ^70^, ^71^, ^72^, ^73^, ^74^, ^75^, ^76^, ^77^, ^78^, ^79^, ^80^, ^81^, ^82^, ^83^, ^84^, ^85^, ^86^ In most of these studies, thrombolytics were administered within 60 minutes of thrombosis,^10^, ^18^, ^26^, ^27^, ^28^, ^29^, ^31^, ^33^, ^34^, ^36^, ^39^, ^40^, ^41^, ^45^, ^46^, ^47^, ^48^, ^49^, ^50^, ^51^, ^52^, ^53^, ^54^, ^55^, ^56^, ^57^, ^58^, ^61^, ^64^, ^65^, ^66^, ^67^, ^68^, ^69^, ^73^, ^74^, ^75^, ^76^, ^78^, ^79^, ^80^, ^81^, ^82^, ^84^, ^85^, ^87^, ^88^, ^89^, ^90^, ^91^, ^92^, ^93^, ^94^ with fewer studies investigating thrombolytics administered 90 minutes,^43^, ^44^, ^86^, ^95^ 2 hours,^30^, ^38^, ^62^, ^72^, ^77^ 3 hours,^63^, ^70^ or 6 hours^96^ after thrombosis.

One case report (LOE 5) also supported the PICO question.^97^ This report was considered to be of fair quality since it had objective outcome measures and long‐term follow‐up. This case report described an 8‐year‐old, intact female Maltese dog that was suspected to be hypercoagulable due to a malignant mammary adenocarcinoma.^97^ The dog presented for evaluation of a 3‐day history of unilateral (right) pelvic limb paralysis, and had a weak femoral pulse. No blood flow was detected in the artery by Doppler, and thermography was used to determine the location of the arterial thrombus. Initial IV administration of streptokinase was ineffective. Subsequent local, intraarterial, catheter‐directed administration of streptokinase was effective; vessel patency was confirmed by thermography, and the right femoral pulse was palpable. Complications associated with hemorrhage or reperfusion were not evident. UFH and clopidogrel were also administered, and the dog was discharged with a near normal gait on the third day following thrombolysis. At 10‐month follow‐up, thrombosis had not recurred, and the dog was still receiving clopidogrel.^97^

Another case report was considered neutral to the PICO question, since rt‐PA was given early in the treatment course.^98^ This case report described a 5.3‐year‐old male Yorkshire Terrier with distal aortic thrombosis secondary to protein‐losing enteropathy. IV rt‐PA was administered (1 mg/kg bolus every 60 min for a total of 10 doses), after which pelvic limb withdrawal responses returned, and pulses improved. Due to a stagnation in clinical improvement, 2 additional doses of rt‐PA were administered on each of the sixth and seventh days of hospitalisation; pulse quality was again noted to improve, no complications were evident, and the dog was discharged on the ninth day.^98^

No studies were identified that opposed the worksheet question.

### 3.2 PICO question: Thrombolytic agents in arterial thrombosis (dogs)

In dogs with suspected or confirmed arterial thrombosis (P), does use of 1 specific thrombolytic agent (I) compared to any other thrombolytic agent (C) improve any outcomes (O)?

#### 3.2.1 Guidelines

##### Thrombolytic agents in arterial thrombosis (dogs)

Delphi consensus reached in 19/19, Round 1

- In dogs with confirmed acute arterial thrombosis, there is insufficient evidence to support the use of one thrombolytic agent over another.
- Of the currently available thrombolytic drugs, rt‐PA has been used most widely, but when indicated the choice of thrombolytic agent will likely be dictated by availability.

#### 3.2.2 Evidence summary

Ten studies supported the PICO question (all LOE 3, 3 good quality,^56^, ^75^, ^99^ 6 fair,^16^, ^17^, ^32^, ^53^, ^74^, ^77^ 1 poor^35^), 2 were neutral (both LOE 3, fair),^20^, ^23^ and none opposed the question. Nonetheless, there was little consistency in findings across the studies supporting the PICO question precluding any conclusions supporting one drug over another. The vast majority of the experimental literature of arterial thrombolysis in dogs involved the use of rt‐PA and lacked comparisons with other commercially available thrombolytics.^9^, ^13^, ^19^, ^24^, ^25^, ^28^, ^29^, ^30^, ^31^, ^33^, ^36^, ^38^, ^39^, ^40^, ^41^, ^42^, ^43^, ^44^, ^45^, ^46^, ^47^, ^48^, ^51^, ^52^, ^54^, ^55^, ^57^, ^58^, ^59^, ^61^, ^63^, ^64^, ^67^, ^68^, ^69^, ^70^, ^71^, ^73^, ^76^, ^78^, ^79^, ^80^, ^82^, ^83^, ^84^, ^85^, ^86^, ^87^, ^88^, ^89^, ^90^, ^92^, ^93^, ^94^, ^95^, ^100^, ^101^, ^102^

Of the 10 supporting studies, 4 demonstrated that a third‐generation thrombolytic agent was superior to a second‐generation thrombolytic. Specifically, 2 studies supported that monteplase was superior to rt‐PA,^53^, ^56^ 1 noted that intraarterial reteplase was superior to IV alteplase,^35^ and 1 that noted that IV reteplase was superior to IV alteplase.^74^ One study demonstrated that a secondary generation thrombolytic (alteplase) and a third‐generation thrombolytic (reteplase) were both superior to the first‐generation drug streptokinase.^99^ Interestingly, although 3 studies demonstrated that alteplase was superior to the first generation drugs urokinase or single‐chain urokinase,^16^, ^17^, ^75^ other studies suggested superiority of urokinase^77^ or pro‐urokinase^32^ over tPA. Each of the included studies is described in more detail below.

Martin and colleagues (LOE 3, good) demonstrated that alteplase and reteplase were superior to streptokinase.^99^ They randomized dogs to receive 1 of 4 thrombolytic drugs administered 60 minutes after thrombotic occlusion of the left circumflex coronary artery.^99^ Although one of the thrombolytics is not commercially available (a recombinant *Escherichia coli*‐produced protease domain of tPA), the other 3 groups (alteplase, reteplase, and streptokinase) are clinically relevant. Thrombolytic doses were as follows: streptokinase (21,000 IU/kg as a 60‐min IV infusion), reteplase (double bolus injection, 0.14 U/kg [0.24 mg/kg] over 2 min, repeated 30 min later), and alteplase (3 step infusion to achieve a total dose of 1.45 mg/kg over 90 min). The alteplase dose was given as an IV bolus of 0.2 mg/kg over 2 minutes, followed by a 0.75 mg/kg infusion over 28 minutes, then a 0.5 mg/kg infusion over 60 minutes. Reperfusion rates at 180 minutes after the onset of thrombolysis were similar across groups (8/8 alteplase, 8/8 reteplase, and 7/8 streptokinase), but cumulative patency time was significantly longer in the reteplase group compared to the streptokinase group. Additionally, the postmortem weight of the residual thrombus was significantly lower in the alteplase and reteplase groups than the streptokinase group. Regarding adverse effects, the incidence of rebleeding from 1‐ and 2‐day‐old ear incision sites was highest in the alteplase group, but differences were not statistically significant.^99^

Saito and colleagues (LOE 3, good) demonstrated superiority of monteplase to native t‐PA and urokinase. They randomized dogs (*n* = 6/group) to receive either monteplase (E6010), native t‐PA, or urokinase 60 minutes after LAD coronary artery occlusion.^56^ Monteplase was administered as a single IV bolus (0.2 mg/kg).^56^ The total t‐PA dose was 0.6 mg/kg, with 10% administered as a bolus and the remaining 90% administered as an IV CRI over 60 minutes. The total dose of urokinase was 60,000 IU/kg administered as an IV CRI, again with 10% of the dose given as a bolus and the remaining 90% over 1 hour. Time to complete reperfusion was not different among groups. The rate of reperfusion, however, was more gradual in the monteplase group, than the t‐PA or urokinase groups (*P* < 0.01). Reperfusion with native t‐PA and urokinase resulted in significantly more ventricular premature contractions per minute compared to baseline; however, this was not evident in the monteplase group. Monteplase‐treated dogs also had significantly fewer ventricular premature contractions at 15 minutes after reperfusion than the urokinase group (*P* < 0.05). While mortality due to VF was not statistically different between groups, it was numerically higher in the t‐PA (3/6) and urokinase (2/6) groups, than the monteplase group (0/6). As such, monteplase was considered superior to native t‐PA and urokinase in this study.^56^

The same group compared the effects of monteplase‐induced thrombolysis to rt‐PA and urokinase on left ventricular function in the same model of coronary artery thrombosis (LOE 3, fair); again demonstrating some evidence for superiority of monteplase.^53^ Thrombolysis was commenced 30 minutes after occlusion with either monteplase (0.2 mg/kg IV bolus), rt‐PA (0.6 mg/kg total dose, 10% bolus, then 90% as IV CRI over 1 h), or urokinase (0.38 mg/kg IV CRI over 1 h). Reperfusion time was not significantly different among groups, and no reocclusion was observed over a 4‐hour period. Monteplase was superior to rt‐PA and urokinase with regard to earlier recovery of left ventricular ejection fraction and regional wall motion.^53^

Interestingly, another study by the same group (LOE 3, fair), this time using a femoral artery thrombosis model, was considered neutral to the PICO question in that monteplase had equivalent thrombolytic activity to an IV CRI of rt‐PA at the same dose.^20^

Gurewich and colleagues (LOE 3, fair) compared IV administration of rt‐PA with the M5 mutant of pro‐urokinase in a femoral artery thrombosis model.^32^ Pro‐urokinase (2 mg/kg IV) was comparably effective to rt‐PA (1.4 mg/kg IV over 60 min, with 20% given as a bolus) when assessed by radioisotope counts over the femoral artery at 90 minutes after therapy, as well as postmortem examination. Rethrombosis developed in 1 dog in the rt‐PA group. Safety was superior in the pro‐urokinase group, with much lower blood loss from standardized incisions on the abdomen and ear (mean: ∼4 ml blood loss) than the rt‐PA group (mean: 40 ml, *P* = 0.026). One dog in the rt‐PA group lost >110 ml blood (>10% body weight).^32^

Qureschi and colleagues (LOE 3, poor) performed a randomized comparison of intraarterial reteplase to IV alteplase in a dog model of acute basilar artery thrombosis.^35^ It was considered of poor quality to answer the PICO question because although 2 different thrombolytic drugs were compared in a randomized fashion, they were administered by different routes. Thrombolysis was performed 2 hours after verification of arterial occlusion, and serial angiography was used to monitor for recanalization over a 6‐hour period. Additionally, a postmortem brain MRI was performed to assess for hemorrhage or infarction. Alteplase was administered at a dose of 0.9 mg/kg infused over 90 minutes IV, compared to catheter‐directed reteplase injected into the proximal portion of the clot (0.09 U/kg over 20 min). The reteplase dose is considered to be equivalent to half the alteplase dose. At 6 hours, there was no significant difference in the rates of partial or complete recanalization between groups (2/6 in the alteplase group, 5/7 in the reteplase group, *P* = 0.2), and reocclusion occurred in 1 dog in each group. Intraarterial reteplase was considered superior since it was not associated with intracerebral hemorrhage (0/7) in contrast to IV alteplase (4/6, *P* = 0.02), including 1 dog that experienced hemorrhagic transformation of the cerebellar infarct.^35^

Maki and colleagues compared the catheter‐directed intracoronary administration of urokinase (480,000 units, *n* = 6) with tPA (alteplase, 12,000,000 units, *n* = 8) 30 minutes after the formation of an occlusive thrombus in the LAD coronary artery of dogs (25–30 kg body weight).^16^ Alteplase was superior to urokinase in that all dogs in the alteplase group experienced complete thrombolysis when assessed angiographically and angioscopically 60 minutes after treatment. In contrast, thrombolysis was incomplete in the urokinase‐treated dogs, and angioscopy demonstrated residual adherent thrombi in all dogs.^16^

Haberstroh and colleagues compared the thrombolytic efficacy of local versus systemic thrombolysis with urokinase, scu‐PA, and rt‐PA in a canine model of renal artery thrombosis in which all dogs were systemically heparinized (LOE 3, fair).^17^ This study used Labrador‐Harrier dogs with a mean body weight of 18.9 kg (min–max: 18–25 kg). Systemic thrombolysis resulted in shorter re‐canalization times than local thrombolysis (*P* < 0.01 for each comparison), when compared for each drug and assessed by digital subtraction angiography. Systemic rt‐PA (6.25 mg/h for 150 min, *n* = 6) resulted in shorter recanalization time (mean: 50 ± 12 min) than urokinase (400,000 U/h for 150 min, *n* = 6, 58 ± 22 min) and scu‐PA (1,600,000 U/h for 150 min, *n* = 6, recanalization time 75 ± 33 min). Similarly, local rt‐PA (1.5 mg/h for 150 min, *n* = 12) resulted in a shorter recanalization time (mean: 68 ± 28 min) than either scu‐PA (100,000 U/h over 150 min, *n* = 12, 80 ± 45 min) or urokinase (30,000 U/h, *n* = 12, 102 ± 30 min). Nonetheless, kidney function was adversely compromised at 8‐week follow‐up in all dogs, even after successful thrombolysis.^17^

Nicolini and colleagues (LOE 3, fair) evaluated equimolar doses of rt‐PA (1 mg/kg IV over 20 min) and reteplase (K2P, 0.65 mg/kg IV over 20 min) administered after 30 minutes of stable coronary artery thrombosis.^74^ There was no significant difference in occurrence of reflow (6/10 rt‐PA vs 5/5 reteplase), time to reflow, or mean peak reflow rate between groups. However, mean coronary artery flow at 60 minutes after reperfusion was greater in the reteplase group (49 ± 16 ml/min) than the tPA group (7 ± 3 ml/min, *P* < 0.02). Additionally, although the frequency of reperfusion‐induced ventricular arrhythmias was not quantified, no dogs in the reteplase group required lidocaine treatment, while lidocaine was required in the rt‐PA‐treated dogs. Nonetheless, despite thrombolysis, neither thrombolytic restored normal left ventricular myocardial function at 1 hour after reperfusion.^74^

Gu and colleagues demonstrated superiority of catheter‐directed intracoronary administration of rt‐PA compared to urokinase in a coronary artery thrombosis model (LOE 3, good).^75^ All thrombolytics were infused over 45 minutes. Specifically, 0.75 mg/kg of rt‐PA resulted in the greatest rate and extent of coronary thrombolysis measured by decay of the radiolabeled thrombus, when compared to 0.25 mg/kg rt‐PA, 15,000 U/kg urokinase, and 30,000 U/kg urokinase (*P* < 0.05). Additionally, the lower rt‐PA dose resulted in a greater thrombolysis than the lower urokinase dose (*P* < 0.05).

Although some studies have shown that rt‐PA is superior to urokinase, a study by Fitzgerald and colleagues documented the opposite in a coronary artery occlusion model (LOE 3, fair).^77^ In their study, groups a, c, d, and f were most relevant to the PICO question, and they compared rt‐PA to urokinase and pro‐urokinase. In each group, the thrombolytic was commenced 2 hours after complete coronary occlusion and administered as a CRI via a peripheral vein until 10 minutes after reperfusion. The rt‐PA rate was 10 μg/kg/min (*n* = 10). Urokinase was administered at either 1000 IU/kg/min (*n* = 8) or as a 6600 IU/kg bolus, followed by an infusion of 75 IU/kg/min (*n* = 12); these groups were combined for analysis given that reperfusion rates were not different. Pro‐urokinase was administered at 20 μg/kg/min (*n* = 7). Time to reperfusion was similar for rt‐PA, urokinase, and pro‐urokinase; however, the rate of complete reocclusion was significantly higher in the rt‐PA group (9/10) than the urokinase group (1/20, *P* < 0.001). Nonetheless, cyclic flow variations did occur in the urokinase groups. The rate of bleeding from a standardized thoracic incision was not different among groups.^77^

Another study was neutral to the PICO question, demonstrating no difference in efficacy between comparable doses (0.25, 0.5, and 1 mg/kg) of thrombolytic (rt‐PA and rscu‐PA) as measured by frequency and rate of recanalization and persistence of patency over 2 hours, in a femoral artery eversion graft model of thrombosis.^23^

### 3.3 PICO question: Thrombolysis in venous thrombosis (dogs)

In dogs with suspected or confirmed venous thrombosis (P), does use of a thrombolytic agent (I) compared to no thrombolytic agent (C) improve any outcomes (O)?

#### 3.3.1 Guidelines

##### Thrombolysis in venous thrombosis (dogs)

Delphi consensus reached in 16/16, Round 2

- In dogs with confirmed acute venous thrombosis, we suggest use of a thrombolytic agent can be considered following an assessment of the risk and benefit in individual patients.
- We suggest the thrombolytic agent be delivered in a catheter‐directed manner if feasible.
- No evidence‐based recommendations can be made regarding the use of thrombolytic agents for treatment of chronic venous thrombosis in dogs.

#### 3.3.2 Evidence summary

Four experimental studies (LOE 3, 1 good,^103^ 2 fair^23^, ^104^, ^105^) demonstrated improved outcomes with the use of a thrombolytic agent versus placebo in dogs with venous thrombosis. These included 2 combined coronary arterial and femoral venous thrombosis models,^23^, ^103^ a deep vein thrombosis model (femoral veins) using catheter‐directed urokinase,^104^ and a retinal vein thrombosis model using microcatheter‐directed tPA infusion.^105^ Given the experimental nature of these studies, and unusual location of the thrombosis relative to clinically observed venous thrombosis in dogs, the studies may have limited relevance to treatment of naturally occurring venous thrombosis. One case report (LOE 5)^106^ also supported the PICO question. One study was neutral to the PICO question (LOE 3, fair),^107^ while no studies were identified that opposed the PICO question.

Many other experimental studies described the administration of thrombolytics to dogs with venous thrombosis,^23^, ^64^, ^83^, ^108^, ^109^, ^110^, ^111^, ^112^, ^113^, ^114^, ^115^ but lacked a control group and thus did not address the PICO question. There are also reports of the use of thrombolytics in dogs with naturally developing venous thrombosis,^116^, ^117^ although the lack of placebo‐treated dogs precludes their use in addressing the PICO question.

Rapold and colleagues (LOE 3, good) investigated an rscu‐PA (saruplase) in a combined model of arterial and venous thrombosis.^103^ Venous thrombi were allowed to age for 30 minutes before treatment. Dogs were randomized to 1 of 5 groups. Infusion of 1 mg/kg saruplase over 60 minutes (Group I) induced femoral vein recanalization in 4 of 5 dogs with 98% ± 1% (mean ± SEM) venous clot lysis. Bolus injection of 1 mg/kg saruplase (Group II) caused reflow in 3 of 5 dogs with 88% ± 5% venous clot lysis. Infusion of 0.5 mg/kg saruplase over 60 minutes (Group III) achieved reflow in 3 of 5 dogs with 52% ± 6% venous clot lysis. Bolus injection of 0.5 mg/kg saruplase (Group IV) induced reflow in 4 of 5 dogs with 48% ± 12% venous clot lysis. Placebo infusion (Group V) was associated with late recanalization in 1 of 5 dogs with only 18% ± 8% venous clot lysis.^103^ There was no mention of hemorrhagic complications. As such, this study supports the PICO question that thrombolysis with either a bolus dose or IV infusion of thrombolytic is superior to control.

Lu and colleagues (LOE 3, fair) conducted a controlled, nonrandomized study investigating the efficacy of a novel chimera of uPA and tPA with or without an antiplatelet agent (ridogrel), compared with standard rscu‐PA, rt‐PA, or no‐thrombolytic control.^23^ The chimeric molecule is not commercially available, but comparisons of uPA and rt‐PA with the no‐thrombolytic control are clinically relevant. Only the venous thrombosis models were assessed for this PICO question. Thrombolytic infusion was commenced 1 minute after thrombosis induction. Groups of 5 dogs received rscu‐PA at doses of 0.25, 0.5, or 1 mg/kg, or rt‐PA, also at doses of 0.25, 0.5, and 1 mg/kg with ridogrel. Additionally, groups of 3 dogs received 1 mg/kg rscu‐PA or 1 mg/kg rt‐PA without ridogrel, a control group of 5 dogs received no thrombolytic but ridogrel, while another control group of 3 dogs received no thrombolytic and no ridogrel. The degree of clot lysis was lower in the 2 control groups (22% ± 1% without ridogrel, 28% ± 4% with ridogrel) compared to the 1 mg/kg rscu‐PA group (80% ± 6% without ridogrel, 75% ± 6% with ridogrel) and the 1 mg/kg rt‐PA group (84% ± 6% without ridogrel, 78% ± 5% with ridogrel).^23^ This study supports the PICO question in that thrombolysis occurring after treatment with either rscu‐PA or rt‐PA was superior to no thrombolytic treatment in dogs with venous thrombosis.

Cho and colleagues (LOE 3, fair) compared the efficacy of thrombolysis with urokinase to balloon catheter thrombectomy or no treatment in a randomized acute deep venous thrombosis model in dogs.^104^ Efficacy was assessed by duplex ultrasound scanning involving B‐mode and Doppler measurements,^118^ repeated every 30 minutes until complete clot lysis. Urokinase at 4000 U/min (*n* = 5) was administered in a catheter‐directed fashion until complete clot lysis. Three hours after restoration of flow, 1 vein was harvested from each dog for functional ex vivo studies and histologic analysis. The dogs were then injected with radiolabeled platelets and fibrin, and the remaining vein was harvested 3 hours later to assess thrombogenicity. The control group in this experiment (*n* = 6) received no treatment, and the thrombosed veins were not disturbed until removal for thrombogenicity experiments. Clot lysis in the urokinase group and clot removal in the thrombectomy group were successfully achieved in all cases, compared to the group without treatment, where the intraluminal thrombus remained. Clot lysis occurred within 90 minutes in 4 of 5 urokinase‐treated dogs, while the remaining dog required 120 minutes of urokinase infusion for lysis. No dogs in the thrombolysis group experienced recurrence within 3 hours, in comparison to 5 of 9 dogs in the thrombectomy group. Compared to thrombectomy, thrombolysis resulted in better preservation of the endothelial and smooth muscle functional characteristics of the veins and reduced thrombogenicity. This study supports the PICO question in that thrombolysis with urokinase was superior to no treatment. Adverse effects such as bleeding were not noted.^104^

The final experimental study that supports the PICO question (LOE 3, fair) involved a model of experimental retinal vein occlusion in dogs.^105^ Thrombolytic treatment was instituted 1 week after induction of venous occlusion at which time all eyes had severe sequelae of occlusion including intraretinal hemorrhage, edema, and dilated tortuous veins. Four eyes were treated with infusion of rt‐PA via a microcatheter in the retinal vein, while 4 eyes were left untreated. Total rt‐PA doses ranged from 400 to 1000 μg, infused over 25–45 minutes. No intraoperative or postoperative complications were observed. One week after rt‐PA treatment (2 weeks after thrombosis), all treated eyes demonstrated marked improvement in the retinal hemorrhages, edema, retinal vein dilation, and tortuosity that was not evident in the control eyes. Three dogs in each group had follow‐up at 1 month after rt‐PA treatment (5 weeks after thrombosis); all tPA‐treated eyes showed restoration of retinal vein flow, with no evidence of recurrence or stenosis, and complete clearance of the retinal hemorrhage and edema that was not evident in the control eyes. One dog in the rt‐PA treatment group was euthanized 1 week after rt‐PA treatment, and ocular histopathology revealed no signs of thrombosis or retinal vein dilation, with normal surrounding retinal tissue.^105^ Although this study supports the PICO question, its relevance to naturally occurring venous thrombosis in dogs is limited.

Another study using the described femoral vein ligation model was neutral to the PICO question.^107^ Bilateral thrombosis was created by 48 hours of proximal and distal femoral vein ligation, after which dogs were randomized into groups treated with thrombolysis via catheter‐directed urokinase infusion (4000 U/min for 90 min; *n* = 6) or Fogarty balloon catheter thrombectomy (*n* = 6). There was no placebo‐treated group (although there was a sham operated group), and final outcomes were assessed at 4 weeks. The study is considered neutral to the PICO question since there was no placebo‐treated group, and all veins were patent at 90 minutes and 1 month regardless of treatment allocation.^107^ Thrombectomy was inferior to thrombolysis with respect to residual thrombi with 4 of 6 thrombectomy veins (66%) and 1 of 6 (17%) thrombolyzed veins demonstrating residual thrombus at the site.^107^

A case report considered to support the PICO question described a 4‐year‐old male neutered Maltese dog that developed cranial vena cava thrombosis associated with an indwelling central venous catheter placed during hospitalization for treatment of polytrauma and secondary sepsis.^98^ Over a period of 4 days, the dog's condition progressed from having a palpably thickened jugular vein to a chylothorax, and ultimately cervical and abdominal subcutaneous edema. Occlusive thrombosis of the cranial vena cava was confirmed by venography. Catheter‐directed rt‐PA was administered via the right jugular vein, for a total of 4 doses of 0.4 mg/kg rt‐PA at 60 minutes intervals. This therapy resulted in hemorrhage (estimated 70–80 ml of blood loss) but a reduction in thrombus size (demonstrated sonographically). A second course of rt‐PA (0.2 mg/kg IV, q 60 min for 5 doses) resulted in a further reduction in thrombus size and further hemorrhage (50 ml) that resolved spontaneously within 90 minutes of cessation of thrombolytic infusion. After the second course of treatment, the chylothorax progressively reduced in volume.^106^

### 3.4 PICO question: Thrombolytic agents in venous thrombosis (dogs)

In dogs with suspected or confirmed venous thrombosis (P), does use of 1 specific thrombolytic agent (I) compared to any other thrombolytic agent (C) improve any outcomes (O)?

#### 3.4.1 Guidelines

##### Thrombolytic agents in venous thrombosis (dogs)

Delphi consensus reached in 19/19, Round 1

- In dogs with confirmed venous thrombosis, there is insufficient evidence to support the use of one thrombolytic agent over another.
- Of the currently available thrombolytic drugs, rt‐PA has been used most widely, but when indicated the choice of thrombolytic agent will likely be dictated by availability.

#### 3.4.2 Evidence summary

Only 2 experimental studies (LOE 3, fair) were identified that directly compared 2 commercially available thrombolytics (urokinase and rt‐PA) in dogs, both were considered neutral to the PICO question in that the effects of urokinase and rt‐PA were similar.^23^, ^110^ Studies evaluating dual‐agent thrombolysis versus a single thrombolytic agent were excluded.^112^, ^114^, ^115^ Other studies evaluated novel products not commercially available,^108^, ^109^, ^113^, ^119^, ^120^ and thus were not considered in the final guideline recommendation.

Valji and colleagues (LOE 3, fair) investigated the efficacy of intrathrombic compared to parathrombic infusion of urokinase or rt‐PA in a canine model of iliac vein thrombosis.^110^ This study was considered neutral to PICO question in that efficacy of urokinase and rt‐PA was similar, but intrathrombic injection was more effective than parathrombic infusion.

Lu and colleagues investigated a novel chimera of uPA and tPA, with standard rscu‐PA or rt‐PA.^23^ Although the chimeric molecule is not commercially available, the comparison between uPA and rt‐PA informs the PICO question. The model used was a combined model of arterial and venous thrombosis, with the venous component informing this PICO question. Thrombolytic infusion was commenced 1 minute after thrombus induction. Groups of 5 dogs received rscu‐PA at doses of 0.25, 0.5, or 1 mg/kg, or rt‐PA, also at doses of 0.25, 0.5, and 1 mg/kg with the antiplatelet agent ridogrel. Additional groups of 3 dogs each received 1 mg/kg rscu‐PA or 1 mg/kg rt‐PA without ridogrel. A dose‐dependent increase in lysis of the femoral vein thrombi occurred with no difference between rscu‐PA and rt‐PA groups. At the 1 mg/kg dose, total clot lysis was 80% ± 6% in the rscu‐PA and rt‐PA groups, compared to 22%–28% ± 1%–4% in the control groups without and with ridogrel, respectively.^23^

### 3.5 PICO question: Anticoagulants with thrombolysis (dogs)

In dogs with suspected or confirmed venous or arterial thrombosis (P), does use of a combination of an anticoagulant and a thrombolytic agent (I) compared to use of a thrombolytic agent alone (C) improve any outcomes (O)?

#### 3.5.1 Guidelines

##### Anticoagulants with thrombolysis (dogs)

Delphi consensus reached in 19/19, Round 1

- We suggest that combining an anticoagulant with a thrombolytic agent can be considered for treatment of dogs with confirmed arterial or venous thrombosis, where other risk factors for thrombosis exist.
- Strong evidence for improved patient‐centered outcomes is lacking and careful consideration of the potential increased risk of bleeding is indicated.
- No evidence‐based recommendations can be made with respect to the timing of anticoagulant administration in dogs undergoing thrombolysis.

#### 3.5.2 Evidence summary

Thirteen studies support the PICO question (all LOE 3),^40^, ^46^, ^48^, ^51^, ^64^, ^65^, ^71^, ^73^, ^80^, ^83^, ^85^, ^89^, ^92^ while 3 were neutral (LOE 3), and none opposed it. Most studies were coronary artery thrombosis models, in addition to studies modeling thrombosis of the carotid artery,^48^ femoral artery,^85^ a combination of femoral artery and femoral vein,^64^, ^83^ and 1 study of pulmonary embolism.^121^ These studies vary in the anticoagulants used, including UFH, low‐molecular‐weight heparin (LMWH), factor Xa inhibitors, and direct thrombin inhibitors. Additionally, the timing of administration or commencement of the anticoagulant therapy varies from immediately before, concurrent with, or immediately after the thrombolytic agent. Outcome measures varied, but most common was incidence of re‐thrombosis/re‐occlusion, and some also documented improved reperfusion. Since all were experimental studies, none assessed patient‐centered outcomes such as return of function or survival. Additionally, although many report the effects of the addition of an anticoagulant on coagulation test results, few describe clinical bleeding. Studies were excluded if they lacked a study group that did not receive anticoagulant, or if they investigated combined anticoagulant and antiplatelet agents without a group that received anticoagulant therapy alone.^37^, ^49^

Voytik and colleagues (LOE 3, good) evaluated the effects of 2 doses of UFH compared to saline on reocclusion after rt‐PA‐induced thrombolysis in a dog model of femoral artery thrombosis.^85^ After confirmation of clot lysis, dogs were randomly allocated (*n* = 10/group) to receive an additional dose of rt‐PA (0.4 mg/kg/h, IV, over 1 h), saline, low‐dose UFH (500 U bolus followed by 250 U/h, IV, for 24 h), or high‐dose UFH (1500 U followed by 500 U/h, for 24 h). Rates of reocclusion were significantly lower in the dogs receiving high‐dose UFH (0/10) compared to those receiving a second dose of rt‐PA (9/10). The rate of reocclusion in the saline group was 6/10. The lower dose of UFH was not associated with a significant reduction in reocclusion (3/10) but may reflect a type II error.^85^

Yao and colleagues (LOE 3, good) evaluated the impact of UFH on rt‐PA‐induced thrombolysis in a coronary arterial thrombosis model in dogs.^92^ In this case, UFH was administered concurrent with rt‐PA dosed as a 40 μg/kg bolus followed by 4 μg/kg/min, IV. UFH was administered IV either as a 200 U/kg bolus (Group IIa) or a 200 U/kg bolus followed by 200 U/kg/h, IV, for 180 minutes after reperfusion. Time to thrombolysis was reduced and time to reocclusion was increased in UFH‐treated dogs. No hemorrhagic complications were noted, but 1 dog in the UFH IV infusion group died from VF 30 minutes after thrombolysis.^92^

Rapold and colleagues (LOE 3, good) also reported that UFH improved outcomes of rt‐PA‐induced thrombolysis in a combined model of femoral arterial and femoral venous thrombosis in dogs.^83^ In this model, all dogs were treated with aspirin (2.8 mg/kg, IV) and received 0.5 mg/kg rt‐PA infused IV over 1 hour. Dogs in 1 group received UFH as a 200 U/kg IV bolus, followed by a 100 U/kg/h IV CRI for 2 hours, while dogs in the other group did not receive UFH. More dogs experienced arterial reperfusion in the group receiving UFH (9/10, with 7 having reperfusion within 30 min, and 2 late reperfusion), compared to the other group (4/10, with 1 dog having reperfusion within 30 min, and 3 dogs later). The percentage of venous clot lysis was greater in the group receiving UFH (81% ± 4%) compared to the other group (49% ± 7%).^83^ This led the authors to conclude that arterial thrombolytic therapy with rt‐PA requires concomitant IV UFH for optimal efficacy, and thus supports the PICO question.

Nicolini and colleagues (LOE 3, good) explored the role of adjunctive therapy using LMWH with rt‐PA in a coronary artery thrombosis model in 14 dogs.^73^ The clots were present for 30 minutes, followed by infusion of rt‐PA at a dose of 1 mg/kg, IV over 20 minutes. At the time of reperfusion, dogs were randomized to receive either LMWH (dalteparin as Fragmin, 75 IU/kg IV bolus, followed by an additional 75 IU/kg given over 90 min, *n* = 6) or an IV infusion of saline (*n* = 8). Dogs were observed for 2 hours after the end of drug infusion for evidence of reocclusion. Dogs receiving LMWH were no more likely to experience reperfusion (6/6) than those receiving saline (6/8) but were less likely to experience reocclusion (1/6, 17%) compared to saline (4/6). Although the addition of dalteparin to rt‐PA resulted in a greater prolongation of activated partial thromboplastin time (aPTT) compared to saline, no clinical bleeding was noted in the short observation period.^73^ This study supports the PICO question that the use of LMWH reduces reocclusion after rt‐PA‐induced thrombolysis, and suggests that it may increase the likelihood of reperfusion.

Rigel and colleagues evaluated both UFH and disulfatohirudin (a direct thrombin inhibitor) as potential adjuncts to streptokinase‐induced thrombolysis in a model of coronary artery thrombosis in dogs.^65^ Although not a commonly used anticoagulant in veterinary medicine, hirudin and its derivatives are variably commercially available.^122^ Anticoagulant protocols were commenced after allowing the occlusive thrombus to age for at least 30 minutes and 15 minutes prior to streptokinase (750,000 U total dose, given IV over 60 min, dog body weight 17–24 kg). Dogs were allocated to 1 of 6 anticoagulant treatment groups (*n* = 8/group): (1) saline placebo, (2) low‐dose hirudin (0.3 mg/kg IV bolus followed by 0.3 mg/kg/h, IV), (3) medium‐dose hirudin (1 mg/kg IV bolus followed by 1 mg/kg/h, IV), (4) high‐dose hirudin (2 mg/kg IV bolus followed by 2 mg/kg/h, IV), (5) low‐dose UFH (60 U/kg IV bolus followed by 40 U/kg/h, IV), and (6) high‐dose UFH (100 U/kg IV bolus followed by 60 U/kg/h, IV). Vessel patency was monitored for 180 minutes after initiation of the streptokinase infusion. No reperfusion occurred in any of the saline‐treated dogs, and infusion of any anticoagulant resulted in a reduced mean thrombus mass compared to saline. Anticoagulant administration resulted in a dose‐dependent increased likelihood of reperfusion. High‐ and medium‐dose hirudin resulted in 100% and 75% reperfusion, respectively, which was not significantly different from that achieved by high‐dose UFH therapy (75%). There was no significant difference in the rate of reocclusion between dogs receiving high‐dose hirudin or high‐dose UFH. Overall, high‐dose hirudin appeared superior to high‐dose UFH with a lower time to reperfusion (33 vs 65 min), and longer initial period of reperfusion (106 vs 46 min). High‐dose UFH resulted in a greater prolongation of aPTT than high‐dose hirudin, but clinical bleeding complications were not reported.^122^ This study supports the PICO question in that both anticoagulants (hirudin and UFH) enhanced thrombolysis in this model.

Haskel (LOE 3, good) randomized dogs to receive either 1 of 2 antiplatelet agents or 1 of 2 anticoagulants, compared to a saline control group, as adjuncts to rt‐PA‐induced thrombolysis (17 μg/kg/min, IV over 60 min, 1 mg/kg total dose) in a coronary artery occlusion model.^80^ The anticoagulants used were disulfatohirudin (1.5 mg/kg IV bolus, followed by 1.5 mg/kg/h, IV) and UFH (150 U/kg IV bolus, followed by 50 U/kg/h, IV). Hirudin therapy was commenced 15 minutes before the start of the rt‐PA infusion, while UFH was commenced after the rt‐PA infusion; both infusions were continued for 90 minutes. Hirudin with rt‐PA shortened the time to recanalization compared with the control group and prevented reocclusion in all 6 dogs treated, whereas UFH did not shorten the time to reperfusion and was less effective than hirudin in preventing reocclusion (5/6 dogs).^80^ Despite differences between agents, this study supports the PICO question that addition of an anticoagulant to rt‐PA‐induced thrombolysis improves some outcome measures.

Rubsamen and colleagues (LOE 3, fair) also compared the efficacy of a hirudin derivative (PEG‐hirudin) to UFH to prevent early reocclusion in a 4‐hour period after rt‐PA‐induced thrombolysis of carotid artery thrombi.^48^ Concurrent with administration of rt‐PA, dogs received either PEG‐hirudin (0.3 mg/kg IV bolus, followed by 0.15 mg/kg/h, IV) or UFH (0.3 mg/kg IV bolus, followed by 0.3 mg/kg/h, IV). In contrast to the aforementioned study by Rigel,^65^ PEG‐hirudin but not UFH improved the rate of recanalization and prolonged the time to reocclusion compared to saline placebo.^48^, ^65^ Although there was a difference between the 2 anticoagulants, this study nonetheless supports the PICO question that anticoagulants can be used to improve outcomes (in this case, reperfusion and reocclusion) in dogs with arterial thrombosis when used in combination with thrombolytics.

Nicolini and colleagues (LOE 3, good) also evaluated the efficacy of hirudin and a novel factor Xa inhibitor in a coronary artery thrombosis model in dogs.^51^ All dogs were treated with rt‐PA (1 mg/kg IV over 20 min) after 30 minutes of stable clot. Dogs were randomized to receive either saline (*n* = 12, 0.6 ml/min), hirudin (*n* = 6, 20 μg/kg/min), or the Xa inhibitor (*n* = 6) concurrent with rt‐PA, with a 2‐hour observation period from the time of thrombolysis. Reperfusion occurred in 75% of the saline‐treated dogs and 100% of the hirudin‐treated dogs. There was no difference between saline and hirudin in the time to reperfusion (34 ± 4 vs 37 ± 5 min) or the percentage experiencing occlusion (89% saline, 50% hirudin). The only statistically significant difference was a longer time with 100% flow restoration in the hirudin group (20 ± 6 min, compared to 7 ± 2 min in the saline group). As such, the authors concluded that these doses of hirudin delayed but did not prevent rethrombosis.^51^ Nonetheless, this study was considered to provide some evidence in support of the PICO question.

Leadley and colleagues (LOE 3, good) compared various anticoagulants as adjunctive antithrombotic therapy during rt‐PA thrombolysis in a stenosed canine coronary artery thrombus model.^46^ In this model, rt‐PA was administered as a 100 μg/kg IV bolus, followed by 20 μg/kg/min for 60 minutes. Adjunctive therapy (*n* = 10/group), commenced 15 minutes prior to rt‐PA and after 60 minutes of clot aging, included either saline control, enoxaparin (1 mg/kg IV, followed by 30 μg/kg/min CRI), UFH (50 U/kg, then 0.6 U/kg/min CRI), UFH (same dose) + aspirin (5 mg/kg IV once), or hirulog (2 mg/kg, followed by 40 μg/kg/min CRI). The infusions of anticoagulants were each continued for 135 minutes, and blood flow in the affected arteries was monitored for an additional 2 hours. Enoxaparin resulted in a statistically significant improvement in outcome measures including the total minutes of flow (between lysis and reocclusion) and reduction in thrombus mass. Total minutes of flow were significantly higher in the enoxaparin group (143 ± 25 min), in comparison to vehicle (54 ± 25 min), UFH (69 ± 20 min), and heparin plus aspirin (60 ± 23 min). Thrombus mass was also significantly lower in the enoxaparin group (6.0 ± 1.3 mg) than that in the saline group (11.8 ± 3.2 mg, *P* < 0.05). This study provides support for the PICO question in that some outcome measures were improved by the addition of enoxaparin as an anticoagulant to the thrombolytic compared to the thrombolytic alone.

Jun and colleagues (LOE 3, good) also compared various anticoagulants as adjuncts to coronary thrombolysis with alteplase and aspirin in dogs.^55^ After 1 hour of clot aging, all dogs received a single IV bolus dose of aspirin (5 mg/kg) and an rt‐PA bolus (0.1 mg/kg IV alteplase), followed by a CRI (0.01 mg/kg/min for 30 min). Concurrent with the rt‐PA infusion, dogs received their randomly allocated adjunctive treatment (*n* = 10/group). Group I received saline, Group II received UFH (200 IU/kg bolus, then 100 IU/kg/h CRI), Group III low‐dose nadroparin (an LMWH, 100 IU/kg bolus, then 50 IU/kg/h CRI), Group IV medium‐dose nadroparin (200 IU/kg bolus, then 100 IU/kg/h CRI), and Group V high‐dose nadroparin (300 IU/kg bolus, then 150 IU/kg/h CRI). Anticoagulant infusions were continued for 2 hours, at which time the study was terminated. Dogs in the UFH, as well as the medium‐ and high‐dose nadroparin groups, had significantly improved coronary artery patency at 90 and 120 minutes, compared to the saline and low‐dose nadroparin groups (*P* < 0.001), providing support to the PICO question.

Rebello and colleagues (LOE 3, fair) compared enoxaparin to UFH either alone or in combination with a GPIIb‐IIIa receptor antagonist in a canine coronary artery thrombosis model.^40^ Group 1 (vehicle), Group 4 (UFH 60 U/kg IV bolus, followed by 0.7 U/kg/min CRI for 135 min), and Group 6 (enoxaparin, 0.6 μg/kg IV bolus, followed by 6 μg/kg/min CRI for 135 min) were most relevant to the PICO question. Anticoagulants were commenced 15 minutes prior to rt‐PA (100 μg/kg bolus, followed by 20 μg/kg/min CRI for 60 min). A greater proportion of dogs receiving anticoagulants had successful thrombolysis (4/8 UFH, 4/8 enoxaparin) compared to the saline control group (2/8). Additionally, the time to reocclusion was longer in the dogs receiving anticoagulants (36 min enoxaparin, 56 min UFH), compared to the saline group (20 min). This supports the PICO question that use of anticoagulants in addition to thrombolytics improves some outcomes.^40^

Stassen and colleagues (LOE 3, good) also evaluated LMWH (enoxaparin) and UFH as adjuncts to rt‐PA (alteplase) in a combined femoral arterial and femoral venous thrombosis model in dogs.^64^ All dogs were treated with a 5 mg/kg IV bolus of aspirin and 0.5 mg/kg alteplase (with 0.05 mg/kg as an IV bolus, followed by 0.45 mg/kg as an infusion over 1 h). Dogs were randomized to 1 of 7 groups (*n* = 4/group) to receive either saline, enoxaparin (low‐, medium‐, and high‐dose groups, 1.5, 3, and 6 mg/kg, respectively), or UFH (low‐, medium‐, and high‐dose groups, 0.5, 1, and 2 mg/kg, respectively). Fifty percent of the dose of each anticoagulant was administered as a bolus, followed by the remainder as a 2‐hour infusion. Statistically significant improvements in outcomes in the arterial thrombosis model were seen only in the high‐dose anticoagulant groups when compared to control. Specifically, the time to reflow was significantly shorter in the 6 mg/kg enoxaparin group (19 ± 5 min) and 6 mg/kg UFH group (22 ± 5 min) than the saline group (120 ± 36 min) (*P* < 0.03). Additionally, the total time of arterial patency during the 3‐hour observation period was significantly longer in the 6 mg/kg enoxaparin group (140 ± 13 min) and 6 mg/kg UFH group (120 ± 24 min) than the saline group (9 ± 5 min, *P* < 0.01).^64^

Another study by Nicolini (1994, LOE 3, good) assessed the role of the antiplatelet agent (eptifibatide, a GPIIb‐IIIa receptor antagonist) and anticoagulant hirudin in a canine coronary arterial thrombosis model.^89^ All dogs received rt‐PA for thrombolysis (1 mg/kg IV over 20 min) and were randomized to 1 of 4 adjunctive treatment infusions administered over 90 minutes (*n* = 8/group): saline, eptifibatide (5 μg/kg/min), hirudin (20 μg/kg/min), or a low‐dose combination of the 2 agents (2.5 μg/kg/min eptifibatide and 10 μg/kg/min hirudin). This study supports the PICO question in that the administration of hirudin with tPA resulted in a longer period of complete restoration (100% baseline) of coronary blood flow (26 ± 5 min), compared to rt‐PA and saline (5 ± min), although it did not reduce reocclusion.^89^

Three studies were neutral to the PICO question. Roux and colleagues (LOE 3, fair) evaluated the effects of UFH, aspirin, and a synthetic platelet GPIIb‐IIIa receptor antagonist in a model of canine coronary artery thrombosis.^69^ Two of the 6 treatment groups (groups 1 and 2, *n* = 10/group) are relevant to the PICO question, in that group 1 dogs received rt‐PA as a thrombolytic alone (30 μg/kg/min over 60 min), while group 2 received rt‐PA and UFH as a 200 U/kg bolus followed by a 50 U/kg/h CRI over 2 hours. The addition of UFH did not improve the incidence of reperfusion, time to reperfusion, or the reocclusion rate and as such was neutral to the PICO question.^69^

The second neutral study was in a model of pulmonary embolism in dogs.^121^ These authors found that concurrent administration of LMWH (500 IU/kg bolus, then 900 U/kg over 3 h) did not augment thrombolysis induced by rt‐PA (1.5 mg/kg IV over 45 min).^121^ Additionally, a study by Prager and colleagues (LOE 3, good) was considered neutral to the PICO question since only an anticoagulant (hirudin, 1.5 mg/kg IV) combined with an antiplatelet agent (aspirin, 5 mg/kg IV) and not the anticoagulant (hirudin) alone reduced recurrent thrombosis after rt‐PA (1 mg/kg total dose) induced thrombolysis in a canine coronary arterial thrombosis model.^61^

### 3.6 PICO question: Antiplatelet agents with thrombolysis (dogs)

In dogs with suspected or confirmed venous or arterial thrombosis (P), does use of a combination of an antiplatelet agent and a thrombolytic agent (I) compared to use of a thrombolytic agent alone (C) improve any outcomes (O)?

#### 3.6.1 Guidelines

##### Antiplatelet agents with thrombolysis (dogs)

Delphi consensus reached in 19/19, Round 1

- We suggest that combining an antiplatelet agent with a thrombolytic agent can be considered for treatment of dogs with confirmed arterial or venous thrombosis, where other risk factors for thrombosis exist.
- Strong evidence for improved patient‐centered outcomes is lacking and careful consideration of the potential increased risk of bleeding is required.
- No evidence‐based recommendations can be made with respect to the timing of antiplatelet agent administration in dogs undergoing thrombolysis.

#### 3.6.2 Evidence summary

Four studies (all LOE 3) supported the PICO question, 2 of good quality that used the antiplatelet agents abciximab and eptifibatide, respectively,^9^, ^89^ and 2 of fair quality that used clopidogrel and aspirin, respectively.^69^, ^70^ There are also numerous studies of thrombolysis in which all dogs received aspirin at doses of 5 mg/kg IV^40^, ^43^, ^44^, ^45^, ^55^, ^64^ or 20 mg/kg IV^49^, ^99^ prior to thrombolysis or anticoagulation. Additionally, case series and case reports have reported the use of aspirin^116^ or clopidogrel^97^, ^98^ in association with thrombolytics in dogs with naturally occurring thrombosis but did not directly address the PICO question as there was no comparator group. Studies assessing antiplatelet drugs that are not commercially available pharmaceuticals such as ridogrel,^66^ DMP728,^86^, ^123^ CRL42796,^37^ BIBU 52ZW (Fradafiban),^31^ RPR109891,^40^ and TP‐9201^124^ were not included in the systematic review.

Rebello and colleagues (LOE 3, good) investigated the effect of a 0.8 mg/kg IV dose of abciximab (7E3) on reocclusion after successful coronary thrombolysis with rt‐PA.^9^ The administration of abciximab after the completion of a 90‐minute rt‐PA IV infusion was more effective at preventing reocclusion (0/10 reocclusion) than if the same dose was administered at the first evidence of thrombolysis (2/8, *P* < 0.05) or 5 minutes before rt‐PA (5/10, *P* < 0.05). Additionally, the percentage of flow restoration after 6 hours of observation compared to preocclusion values was higher in the abciximab after rt‐PA group (82%), compared to the abciximab before rt‐PA (27%, *P* < 0.001) and abciximab during rt‐PA groups (35%, *P* < 0.003). Regarding arrhythmias, 2 of 12 in the no‐abciximab group, 1 of 14 in the abciximab before rt‐PA, 1 of 10 in the abciximab during rt‐PA, and 2 of 14 in the abciximab after rt‐PA groups experienced mortality due to VF. Additionally, 1 dog in each of the abciximab before and during rt‐PA groups and 2 dogs in the abciximab after rt‐PA group experienced excess bleeding resulting in mortality.^9^ Although the rate of complications was not statistically different among groups, the risk of excess bleeding must be considered.

Nicolini and colleagues (LOE 3, good) found some benefits of the platelet fibrinogen receptor antagonist eptifibatide, particularly when used in combination with the direct thrombin inhibitor hirudin, in a canine coronary artery thrombosis model.^89^ Eptifibatide (5 μg/kg/min for 90 min) or recombinant hirudin (20 μg/kg/min for 90 min) alone improved the magnitude of coronary reflow over time when compared to saline (both *P* < 0.05) but did not affect the rate of reocclusion. When combined at lower doses, eptifibatide (2.5 μg/kg/min for 90 min) and hirudin (10 μg/kg/min for 90 min) resulted in stable and sustained reflow after rt‐PA, significantly reducing the rate of reocclusion.^89^ Prager and colleagues (LOE 3, good) conducted a similar study to that of Nicolini, but it was considered neutral to the PICO question since only the combination of hirudin (1.5 mg/kg IV bolus followed by 1.5 mg/kg/h) and aspirin (5 mg/kg IV bolus), but neither agent alone, shortened the time to reperfusion and reduced recurrent thrombosis after rt‐PA (1 mg/kg total dose) induced thrombolysis in a canine coronary arterial thrombosis model.^61^

Yao and colleagues (LOE 3, fair) demonstrated that clopidogrel (10 mg/kg IV bolus, followed by 2.5 mg/kg/h CRI) was more effective than aspirin (5 mg/kg IV bolus) as an adjunctive treatment when administered concurrently with UFH (200 U/kg) to prevent reocclusion after rt‐PA‐induced lysis of a 3‐hour aged coronary thrombus in dogs.^70^ Specifically dogs receiving clopidogrel by the aforementioned dosing scheduled had no evidence of reocclusion (0/7), compared to 7 of 7 dogs in the aspirin group (*P* < 0.01) and 5 of 5 dogs in the UFH alone group. Additionally, dogs receiving a lower clopidogrel dose (5 mg/kg IV bolus) in addition to UFH had a longer time to reocclusion (153 ± 19 min) than those receiving aspirin and UFH (74 ± 13 min) and UFH alone (72 ± 11 min) (*P* < 0.01).^70^ Clopidogrel use was associated with mild to moderate bleeding around surgical incisions that was subjectively greater in the high‐ versus low‐dose clopidogrel group, and greater than in the aspirin‐treated groups.^70^

In the final study that supported the PICO question, Roux and colleagues (LOE 3, fair) demonstrated some benefit of aspirin (10 mg/kg IV), with or without UFH (200 U/kg followed by 50 U/kg/h), in reducing coronary reocclusion after rt‐PA‐induced thrombolysis in dogs.^69^ This benefit, however, was lost when the thrombogenic stimulus was enhanced.^69^

Five additional studies were neutral to the PICO question. Dommke and colleagues (LOE 3, good) reported that abciximab did not significantly improve clot lysis in dogs treated with the thrombolytic microplasmin after coronary artery thrombosis.^27^ Rote and colleagues (LOE 3, fair) described that pretreatment with the antiplatelet agent ramatroban (BAY U 3405) did not reduce the incidence of coronary artery rethrombosis after anisoylated plasminogen streptokinase activator complex‐induced thrombolysis.^21^ Additionally, Chen and colleagues (LOE 3, poor) reported that aspirin did not potentiate the effects of low‐dose inogatran (a direct thrombin inhibitor) in dogs treated with rt‐PA after coronary artery thrombosis.^52^ Similarly, Leadley and colleagues (LOE 3, good) did not identify differences between dogs receiving UFH alone versus UFH with aspirin (5 mg/kg IV once) with regard to incidence or time to reperfusion, incidence or time to reocclusion, and thrombus mass.^46^ McAuliffe and colleagues (LOE 3, fair) showed no benefit of aspirin over saline placebo in the times to coronary thrombolysis with rt‐PA or rate of reocclusion.^58^

### 3.7 PICO question: Thrombolytic protocols—Alteplase (dogs)

In dogs with suspected or confirmed venous or arterial thrombosis (P), does use of a specific protocol (dose, frequency, route) for use of alteplase (I) compared to any other protocol (C) reduce the risk of complications (eg, fatal or nonfatal hemorrhage) or improve any outcomes (O)?

#### 3.7.1 Guidelines

##### Thrombolytic protocols—Alteplase (dogs)

Delphi consensus reached in 16/16, Round 2

- We suggest that 0.5–1 mg/kg rt‐PA delivered (systemically or catheter‐directed) over 60–90 minutes is associated with successful thrombolysis in dogs with confirmed acute arterial or venous thrombosis.
- There is insufficient evidence to determine if a specific tPA dosing protocol confers a safety benefit.

#### 3.7.2 Evidence summary

Four LOE 3 studies support the PICO question that a specific protocol for rt‐PA improves the chance of successful thrombolysis (2 good quality,^23^ 2 fair quality^125^). One LOE 3 study (good)^88^ and 4 LOE 5 studies^98^, ^106^, ^126^, ^127^ were neutral to the PICO question (discussed in depth in Supporting Information S1).

In a combined model of arterial and venous thrombosis in 75 dogs, Lu and colleagues documented that at least 0.5 mg/kg tPA IV is necessary for consistently successful lysis of femoral arterial thrombi.^23^ More effective thrombolysis of femoral vein thrombi created by injection of whole blood clots was evident at a dose of 1 mg/kg IV (compared to 0.25 or 0.5 mg/kg).^23^

Another LOE 3 good‐quality study demonstrated a dose‐dependent effect of tPA in a canine model of completely occlusive, radiolabeled, femoral arterial thrombosis.^128^ Of note they did not use alteplase specifically, but the product they sourced had equivalent thrombolytic activity to commercially available alteplase. Specifically, 0.10 and 0.20 mg/kg tPA administered IV over 60 minutes resulted in greater thrombolysis (35% and 49%, respectively) than 0.05 mg/kg (15%, *P* < 0.01) as determined by decreased thrombus radioactivity. While clinically relevant efficacy and safety endpoints were not assessed, tPA at these doses did not affect measured prothrombin time, aPTT, thrombin time, hematocrit, platelet count, or fibrin degradation product concentration.^128^

An LOE 3, fair‐quality study evaluated the efficacy of intrathrombic versus parathrombic injection of highly concentrated rt‐PA in dogs in a subacute model of iliac venous thrombosis in 6 dogs (6 additional dogs were treated with urokinase).^110^ Thrombi were created bilaterally such that 1 side could be treated with intrathrombic thrombolytic, while parathrombic infusion was performed on the other side. Intrathrombic injection was performed through a steel catheter, with multiple fenestrations, under high pressure. Thrombi subject to intrathrombic rt‐PA injection all lysed in a median time of 64 ± 26 minutes, while those subject to parathrombic infusion had more variable lysis (3 complete lysis, 1 partial lysis, 2 no lysis).^110^

One LOE 3, fair‐quality study determined that a 60‐minute IV infusion of rt‐PA at 30 μg/kg/min (1.8 mg/kg) was more effective at producing recanalization than 15 μg/kg/min (0.9 mg/kg) in a model of coronary artery thrombosis, with concurrent high‐grade stenosis.^125^ Specifically, 6 of 8 dogs in the high‐dose rt‐PA group achieved recanalization compared to 0 of 4 dogs in the low‐dose group. Nonetheless, 4 of 6 dogs in the high‐dose rt‐PA group experienced reocclusion during or shortly after completing the rt‐PA infusion. The clinical applicability of the model used in this study is limited, however, and the study did not specify what form of tPA was used.^125^

Prewitt and colleagues published an LOE 3 (good) study^88^ in a coronary artery thrombosis model that was neutral to the PICO question. They found no difference in the thrombolytic efficacy of 3 different protocols for the administration of the same total dose of 1.25 mg/kg of IV rt‐PA in heparinized dogs: either a single bolus, 2 boluses administered 15 minutes apart, or a “front loaded” protocol (15% as a bolus, 60% over 30 min, and 25% over 30 min).^88^

### 3.8 PICO question: Thrombolysis in arterial thrombosis (cats)

In cats with suspected or confirmed arterial thrombosis (P), does use of a thrombolytic agent (I) compared to no thrombolytic agent (C) improve any outcomes (O)?

#### 3.8.1 Guidelines

##### Thrombolysis in arterial thrombosis (cats)

Delphi consensus reached in 16/16, Round 2

- No evidence‐based recommendations can be made regarding the use of systemic or catheter‐directed thrombolytic agents for treatment of acute (<6 h) arterial thromboembolism in cats.
- We suggest that thrombolytic agents can be considered for treatment of acute (<6 h) arterial thromboembolism following an assessment of the risk and benefit in individual patients.

#### 3.8.2 Evidence summary

The majority of the literature (11 published manuscripts^129^, ^130^, ^131^, ^132^, ^133^, ^134^, ^135^, ^136^, ^137^, ^138^, ^139^ and 2 abstracts^140^, ^141^) were considered neutral to the PICO question. The studies neutral to the PICO question are summarized in Supporting Information S1. One experimental study (LOE 3, fair)^142^ supported the PICO question, while a historical case series (LOE 5, poor)^143^ opposed the PICO question.

The first report of the use of thrombolytics in cats with arterial thrombosis, published in French in 1969, supported the PICO question albeit with significant quality and clinical relevance limitations (LOE 3, fair).^142^ This study included an experimental study in dogs, an experimental study in cats, and a clinical trial in people.^142^ The experimental components in both dogs and cats involved induction of a femoral arterial thrombus, confirmed by angiography 48 hours later, after which treatment was commenced. Thirteen cats were treated with IV or intraarterial infusions of streptokinase (0.5 ml/min, concentration unclear, total dose of 25,000–100,000 U/h for 6 h), while 5 received saline placebo. Raw data were not presented, but the authors report defibrination, prolongations of prothrombin time/aPTT, and hyperfibrinolytic thromboelastograms. Streptokinase caused clot lysis in 11 of 13 cats, but patient‐centered outcomes such as return of function or survival were lacking.

The study opposing the PICO question, published in Japanese in 2013, was a historical case series of 15 cats with naturally developing aortic thromboembolism (ATE) (LOE 5, poor).^143^ Methodological and reporting limitations reduce the quality of the evidence provided by this study, complicating our interpretation of the information provided. Eight cats (Group A) were treated with LMWH alone (dalteparin 50–200 U/kg IV or SC, q 12–24 h for 1–17 days). Seven cats (Group B) received a combination of dalteparin and monteplase, a third‐generation thrombolytic. Five cats in each group had bilateral pelvic limb involvement, and the majority had concurrent congestive heart failure (5/8 group A, 4/7 group B). Dalteparin doses in group B were 100–200 U/kg IV or SC, every 12 hours for 2–13 days. Monteplase doses were 27,500–72,000 U/kg slow IV or as a CRI given only on day 1. Timing of drug administration relative to the onset of thrombosis was not reported, although timing of hospital presentation after the onset of clinical signs was not different between groups (Group A: 6.5 h [1–60 h], Group B: 2 h [1–8 h]). Adverse events seen in the monteplase group, but not the LMWH group, included hematuria (*n* = 3), anemia (*n* = 1), hyperkalemia (*n* = 1), and a seizure (*n* = 1). Cats treated with monteplase had lower survival to discharge than the LMWH alone group (*P* < 0.05), with only 3 of 7 (43%) surviving to discharge. Of the cats in the LMWH group, 5 were treated as inpatients and all survived to discharge, while 3 were treated as outpatients. While the authors use these data to suggest that monteplase results in worse outcomes, another plausible explanation for the difference in outcome between groups resulted from selection bias (sicker patients in the monteplase group that all required hospitalization).

### 3.9 PICO question: Thrombolytic agents in arterial thrombosis (cats)

In cats with suspected or confirmed arterial thrombosis (P), does use of 1 specific thrombolytic agent (I) compared to any other thrombolytic agent (C) improve any outcomes (O)?

#### 3.9.1 Guidelines

##### Thrombolytic agents in arterial thrombosis (cats)

Delphi consensus reached in 16/16, Round 2

- In cats with confirmed arterial thrombosis, there is insufficient evidence to support the use of one thrombolytic agent over another.
- Of the currently available thrombolytic drugs, tPA has been used most widely for arterial thrombosis in cats, but when indicated the choice of thrombolytic agent will likely be dictated by availability.

#### 3.9.2 Evidence summary

Streptokinase,^129^, ^134^, ^136^, ^142^ urokinase,^132^, ^133^, ^141^ tPA/alteplase,^130^, ^131^, ^135^, ^137^, ^138^, ^139^, ^140^ and monteplase^143^ have all been used for thrombolysis in cats with ATE, but no studies compared thrombolytic agents in this population. The available literature was all therefore considered neutral to the PICO question precluding evidence‐based recommendations regarding the use of one thrombolytic over another in cats with ATE. The CURATIVE Steering Committee and the Domain 6 worksheet authors agree that if thrombolysis is to be pursued, then patient, client, clinician, and pharmacologic factors such as drug availability, drug cost, potential for drug interactions, fibrin specificity, and duration of activity should be considered before making a drug selection. In people with stroke, tPA is the agent of choice for therapeutic thrombolysis,^144^ but the applicability of human stroke guidelines to feline arterial thrombosis is uncertain.

### 3.10 PICO question: Thrombolysis in venous thrombosis (cats)

In cats with suspected or confirmed venous thrombosis (P), does use of a thrombolytic agent (I) compared to no thrombolytic agent (C) improve any outcomes (O)?

#### 3.10.1 Guidelines

##### Thrombolysis in venous thrombosis (cats)

Delphi consensus reached in 16/16, Round 2

- We suggest that in cats with confirmed acute venous thrombosis (<6 h), use of a thrombolytic agent (administered systemically) can be considered when the potential benefits of thrombolysis outweigh the risks of bleeding.

#### 3.10.2 Evidence summary

Two studies (LOE 3, fair)^145^, ^146^ were identified that support the PICO question. No studies that were neutral to or opposed the PICO question were identified.

Picardi and colleagues (LOE 3, fair) created a mesenteric venous thrombosis model in cats and assessed the efficacy of thrombolysis with streptokinase.^145^ Five treatment groups were included but the most relevant to the PICO question were group 4 (*n* = 12 cats) and group 5 (*n* = 11 cats) that underwent reversible clamping of the superior mesenteric vein for 90 minutes, followed by treatment with either LRS (placebo) or streptokinase (90,000 U IV bolus, then 45,000 U/h CRI for 4 h), respectively, commencing 4 hours postoperatively. All cats in the placebo group (group 4) had organized superior mesenteric vein thrombus at postmortem, and 24 hours mortality was 50%. In contrast, cats in the streptokinase‐treated group (group 5) had no evidence of thrombosis at postmortem, and 24 hours mortality was only 18%. Reperfusion injuries or hemorrhagic complications were not reported.^145^

Levinger and colleagues (LOE 3, fair) investigated the use of urokinase in experimentally induced retinal vein occlusion in cats created by laser injury.^146^ One study group (*n* = 15 cats, 28 eyes) received urokinase at a dose of 4000 U/kg IV over 10 minutes followed by 4000 U/kg/h for 4 hours and 50 minutes (total of 5 h), compared to a control group that did not receive urokinase (*n* = 4 cats, 8 eyes). The sooner urokinase was administered after venous clot induction, the more successful it was in resolving the venous occlusion. Specifically, administration of urokinase within 5 minutes (4 eyes) led to similar flow rates through the retinal vein to eyes with no occlusion; however, when urokinase administration was delayed to between 3 and 18 hours, the pressure required to re‐establish flow was 3‐fold greater than in the control eyes. By 24–36 hours postocclusion, even greater pressures were required, and reflow could not be established in some eyes. These data suggest that optimal efficacy of urokinase thrombolysis in this model is within 6 hours of thrombosis.^146^

### 3.11 PICO question: Thrombolytic agents in venous thrombosis (cats)

In cats with suspected or confirmed venous thrombosis (P), does use of 1 specific thrombolytic agent (I) compared to any other thrombolytic agent (C) improve any outcomes (O)?

#### 3.11.1 Guidelines

##### Thrombolytic agents in venous thrombosis (cats)

Delphi consensus reached in 16/16, Round 2

- In cats with confirmed venous thrombosis, there is insufficient evidence to support the use of one thrombolytic agent over another.
- When indicated, the choice of thrombolytic agent will likely be dictated by availability.

#### 3.11.2 Evidence summary

No studies were identified comparing different thrombolytic agents in cats with suspected or confirmed venous thrombosis. The use of streptokinase^145^ and urokinase^146^ has been reported for the treatment of cats with experimentally induced venous thrombosis. Marked differences in efficacy of thrombolysis between these models are reported, but this likely reflects model design, particularly the ability of the thrombolytic drug to reach the thrombus, rather than the thrombolytic agents used.

### 3.12 PICO question: Anticoagulants with thrombolysis (cats)

In cats with suspected or confirmed venous or arterial thrombosis (P), does use of a combination of an anticoagulant and a thrombolytic agent (I) compared to use of a thrombolytic agent alone (C) improve any outcomes (O)?

#### 3.12.1 Guidelines

##### Anticoagulants with thrombolysis (cats)

Delphi consensus reached in 16/16, Round 2

- We suggest that combining an anticoagulant with a thrombolytic agent can be considered for treatment of cats with confirmed arterial thrombosis.
- Strong evidence for improved patient‐centered outcomes is lacking and careful consideration of the potential increased risk of bleeding is required.
- No evidence‐based recommendations can be made with respect to the timing of anticoagulant administration in cats undergoing thrombolysis.
- No evidence‐based recommendations can be made with respect to use of anticoagulants in cats undergoing thrombolysis for venous thrombosis.

#### 3.12.2 Evidence summary

One LOE 5 study (fair)^136^ supported the PICO question, while 8 reports were neutral to the PICO question (1 LOE 4 poor,^139^ 7 LOE 5 poor studies^130^, ^131^, ^133^, ^134^, ^135^, ^136^, ^143^). All identified studies addressed arterial thrombosis, rather than venous thrombosis. The neutral studies are only considered of only fair or poor quality, because they did not directly address the PICO question. Nonetheless, these studies are summarized in Supporting Information S1 since they may inform clinical decision‐making regarding combining anticoagulants with thrombolytics.

Moore et al (LOE 5, fair) published a case series of 46 cats with suspected arterial thrombosis affecting at least 1 limb describing improved survival in those given streptokinase and UFH versus streptokinase alone (*P* = 0.052).^136^ The study was highly relevant to the PICO question but the statistical methods and some relevant results are incompletely described. Streptokinase dosing was not standardized but rather was determined by the clinician and varied widely (bolus of 20,000–25,0000 U, followed by a CRI over 1–28 h at varying doses). The median duration of streptokinase administration was 4 hours, with a median dose of 47,345 U/kg (min–max: 18,857–158,529 U/kg). UFH was administered to some cats, although the number is not stated, at doses from 50 to 232 U/kg every 6 hours SC. The timing of streptokinase or UFH relative to the onset of clinical signs of thrombosis was not described. Overall, 15 of 46 cats survived to hospital discharge; cats treated concurrently with UFH were deemed more likely to survive than those not treated with UFH (*P* = 0.052, which is above the study's a priori threshold).^136^ Additionally, anticoagulants were continued at home in 14 of 15 cats that survived to discharge. Specifically, 12 received coumadin, of which 2 were euthanized after coumadin‐related hemorrhagic complications, and 2 received dalteparin. It is unclear how many cats received UFH and when coumadin or dalteparin was commenced relative to streptokinase or UFH administration.^136^ Of note, the CURATIVE guidelines recommend that other anticoagulants (UFH, LMWH, or direct Xa inhibitors) are used in preference to warfarin/coumadin in cats with thrombosis.^5^

### 3.13 PICO question: Antiplatelet agents with thrombolysis (cats)

In cats with suspected or confirmed venous or arterial thrombosis (P), does use of a combination of an antiplatelet agent and a thrombolytic agent (I) compared to use of a thrombolytic agent alone (C) improve any outcomes (O)?

#### 3.13.1 Guidelines

##### Antiplatelet agents with thrombolysis (cats)

Delphi consensus reached in 16/16, Round 2

- We suggest that combining an antiplatelet agent with a thrombolytic agent can be considered for treatment of cats with confirmed arterial thrombosis.
- Strong evidence for improved patient‐centered outcomes is lacking and careful consideration of the potential increased risk of bleeding is required.
- No evidence‐based recommendations can be made with respect to the timing of antiplatelet agent administration in cats undergoing thrombolysis.
- No evidence‐based recommendations can be made with respect to use of antiplatelet agents in cats undergoing thrombolysis for venous thrombosis.

#### 3.13.2 Evidence summary

Two case series (LOE 4,^139^ LOE 5^136^) and 3 case reports^131^, ^133^, ^134^ describe the use of antiplatelet agents in cats that also received thrombolytic agents for the treatment of ATE. All 5 references are considered neutral since they do not directly address the PICO question. No reports could be found that describe the concurrent use of antiplatelet agents with thrombolytics in cats with venous thrombosis.

The timing of the antiplatelet agents relative to thrombolytics in the case series is unclear, but it appears that antiplatelet agents were commenced either before or concurrently with thrombolytics in each of the case reports. These studies are briefly described here since they provide some evidence of the way in which antiplatelet agents have been used in combination with thrombolytics, which may inform clinical decision‐making.

All of the tPA‐treated cats and most of the standard‐of‐care‐treated cats in the case control study by Guillaumin et al received antiplatelet agents.^139^ Cats in the tPA group (*n* = 16) received either clopidogrel as the sole antiplatelet agent (9) or clopidogrel and aspirin (7). Since this was not a focus of the study, doses were not reported.^139^ Similarly, the concurrent use of antiplatelet agents with thrombolytics was not a focus of the historical case series by Moore et al, although 3 of 15 cats that survived to discharge received aspirin (dose not reported).^136^ Hemorrhage during hospitalization was reported as a complication in some cats in the latter case series; however, it was unclear what antiplatelet medications the affected cats were receiving at the time of hemorrhage. Individual case reports describe 1 cat that received aspirin (25 mg/kg PO q 72 h) with tPA and UFH,^131^ 1 cat that received dipyridamole (12.5 mg PO q 12 h) with urokinase, UFH, and warfarin,^133^ and 1 that received clopidogrel (18.75 mg PO q 24 h) with streptokinase and dalteparin. Hemorrhage was not reported in any of these 3 cases.

### 3.14 PICO question: Thrombolytic protocols—Alteplase (cats)

In cats with suspected or confirmed venous or arterial thrombosis (P), does use of a specific protocol (dose, frequency, route) for use of alteplase (I) compared to any other protocol (C) reduce the risk of complications (eg, fatal or nonfatal hemorrhage) or improve any outcomes (O)?

#### 3.14.1 Guidelines

##### Thrombolytic protocols—Alteplase (cats)

Delphi consensus reached in 16/16, Round 2

- No evidence‐based recommendations can be made for a specific protocol for use of alteplase in cats.

#### 3.14.2 Evidence summary

One case control study (LOE 4),^139^ 3 case series (LOE 5),^130^, ^137^, ^138^ and 3 case reports (LOE 5)^130^, ^131^, ^135^ are considered neutral to the PICO question since they did not compare different alteplase dosing protocols. All used rt‐PA intravenously for treatment of ATE (none described catheter‐directed thrombolysis), but the protocol for alteplase administration varied.

Guillaumin and colleagues used a 1 mg/kg IV rt‐PA dose over 1–1.5 hours but reported slightly different dosing approaches within their population of 16 cats with ATE.^139^ Most commonly, rt‐PA was administered as a 1 mg/kg dose IV over 1 hour (11 cats), but lesser numbers of cats received a 0.1 mg/kg IV loading dose over 1 minutes followed by 0.9 mg/kg IV over 1–1.5 hours (4 cats) or a progressively increasing infusion rate (17% of a 1 mg/kg dose over 1 min, 46% over 30 min, and 37% over 1 h; 1 cat). Despite these minor differences, the total dose was the same (1 mg/kg), with only minor variation in the duration of infusion (1–1.5 h). Based on the small sample size, it was appropriate that cats receiving the different dosing protocols of rt‐PA within this study were not compared.^139^

Welch and colleagues designed a single‐center prospective case series to evaluate the clinical response and side effects of 2 dosing protocols of alteplase in cats with ATE, but terminated the study early due to adverse effects and thus the published results did not answer the PICO question.^137^ Eleven cats were randomized to receive alteplase within 1 hour of presentation, in either group A (5 mg/cat alteplase as an IV CRI over 4 h) or group B (5 mg/cat over 1.5 h; divided as an initial 1 mg IV bolus, then 2.5 mg IV over 30 min, and the remaining 1.5 mg IV over 1 h). Per the study protocol, cats were also permitted to receive an additional dose of tPA (5 mg IV over 4 h) if 1 or more limbs continued to have no pulse or motor function after the initial infusion, and this was done in 4 cats total (2 Group A, 2 Group B). Since body weight was not reported, the milligram per kilogram dose could not be determined but may have been higher than in the study by Guillaumin et al. if some cats weighed <5 kg and in those cats that received a second infusion. As described by the authors, and based on the small sample size, it was appropriate that outcome and adverse events in cats receiving the different dosing protocols of rt‐PA were not compared. Adverse effects were reported in all 11 cats.^137^ It is unclear whether or not the less favorable outcomes in this study compared to that by Guillaumin et al may have been due to higher tPA doses, longer duration of tPA infusion, or other factors such as a longer interval between onset of ATE and administration of tPA.

Much greater variation in doses of rt‐PA has been described in other case reports.^130^, ^140^

## 4 CONCLUSIONS

Generation of guidelines for the use of thrombolytics in dogs and cats is hampered by overall low levels of evidence in the literature. Substantial additional research is needed to address the role of thrombolytics for the treatment of arterial and venous thrombosis in dogs and cats. Clinical trials with patient‐centered outcomes will be most valuable for addressing knowledge gaps in the field.

## CONFLICT OF INTERESTS

Benjamin M. Brainard and Daniel L. Chan are editors of the Journal. The authors declare no other conflict of interest.

## Supporting information

Supporting information

## APPENDIX A GUIDELINES OF THE 2022 CONSENSUS ON THE RATIONAL USE OF ANTITHROMBOTICS AND THROMBOLYTICS IN VETERINARY CRITICAL CARE (CURATIVE): DOMAIN 6—DEFINING RATIONAL USE OF THROMBOLYTICS

### PICO question: Thrombolysis in arterial thrombosis (dogs)

In dogs with suspected or confirmed arterial thrombosis (P), does use of a thrombolytic agent (I) compared to no thrombolytic agent (C) improve any outcomes (O)?

#### Guidelines

##### 1. Thrombolysis in arterial thrombosis (dogs)

- In dogs with confirmed acute arterial thrombosis, particularly where the agent can be delivered within 1 hour of onset of thrombosis, we suggest catheter‐directed intraarterial administration of a thrombolytic agent.
- There is insufficient evidence to determine if thrombolysis improves patient‐centered outcomes.
- No evidence‐based recommendations can be made regarding the use of thrombolytic agents for treatment of chronic arterial thrombosis in dogs.

### PICO question: Thrombolytic agents in arterial thrombosis (dogs)

In dogs with suspected or confirmed arterial thrombosis (P), does use of 1 specific thrombolytic agent (I) compared to any other thrombolytic agent (C) improve any outcomes (O)?

#### Guidelines

##### 2. Thrombolytic agents in arterial thrombosis (dogs)

- In dogs with confirmed acute arterial thrombosis, there is insufficient evidence to support the use of one thrombolytic agent over another.
- Of the currently available thrombolytic drugs, rt‐PA has been used most widely, but when indicated the choice of thrombolytic agent will likely be dictated by availability.

### PICO question: Thrombolysis in venous thrombosis (dogs)

In dogs with suspected or confirmed venous thrombosis (P), does use of a thrombolytic agent (I) compared to no thrombolytic agent (C) improve any outcomes (O)?

#### Guidelines

##### 3. Thrombolysis in venous thrombosis (dogs)

- In dogs with confirmed acute venous thrombosis, we suggest use of a thrombolytic agent can be considered following an assessment of the risk and benefit in individual patients.
- We suggest the thrombolytic agent be delivered in a catheter‐directed manner if feasible.
- No evidence‐based recommendations can be made regarding the use of thrombolytic agents for treatment of chronic venous thrombosis in dogs.

### PICO question: Thrombolytic agents in venous thrombosis (dogs)

In dogs with suspected or confirmed venous thrombosis (P), does use of 1 specific thrombolytic agent (I) compared to any other thrombolytic agent (C) improve any outcomes (O)?

#### Guidelines

##### 4. Thrombolytic agents in venous thrombosis (dogs)

- In dogs with confirmed venous thrombosis, there is insufficient evidence to support the use of one thrombolytic agent over another.
- Of the currently available thrombolytic drugs, rt‐PA has been used most widely, but when indicated the choice of thrombolytic agent will likely be dictated by availability.

### PICO question: Anticoagulants with thrombolysis (dogs)

In dogs with suspected or confirmed venous or arterial thrombosis (P), does use of a combination of an anticoagulant and a thrombolytic agent (I) compared to use of a thrombolytic agent alone (C) improve any outcomes (O)?

#### Guidelines

##### 5. Anticoagulants with thrombolysis (dogs)

- We suggest that combining an anticoagulant with a thrombolytic agent can be considered for treatment of dogs with confirmed arterial or venous thrombosis, where other risk factors for thrombosis exist.
- Strong evidence for improved patient‐centered outcomes is lacking and careful consideration of the potential increased risk of bleeding is indicated.
- No evidence‐based recommendations can be made with respect to the timing of anticoagulant administration in dogs undergoing thrombolysis.

### PICO question: Antiplatelet agents with thrombolysis (dogs)

In dogs with suspected or confirmed venous or arterial thrombosis (P), does use of a combination of an antiplatelet agent and a thrombolytic agent (I) compared to use of a thrombolytic agent alone (C) improve any outcomes (O)?

#### Guidelines

##### 6. Antiplatelet agents with thrombolysis (dogs)

- We suggest that combining an antiplatelet agent with a thrombolytic agent can be considered for treatment of dogs with confirmed arterial or venous thrombosis, where other risk factors for thrombosis exist.
- Strong evidence for improved patient‐centered outcomes is lacking and careful consideration of the potential increased risk of bleeding is required.
- No evidence‐based recommendations can be made with respect to the timing of antiplatelet agent administration in dogs undergoing thrombolysis.

### PICO question: Thrombolytic protocols—Alteplase (dogs)

In dogs with suspected or confirmed venous or arterial thrombosis (P), does use of a specific protocol (dose, frequency, route) for use of alteplase (I) compared to any other protocol (C) reduce the risk of complications (eg, fatal or nonfatal hemorrhage) or improve any outcomes (O)?

#### Guidelines

##### 7. Thrombolytic protocols—Alteplase (dogs)

- We suggest that 0.5–1 mg/kg rt‐PA delivered (systemically or catheter‐directed) over 60–90 minutes is associated with successful thrombolysis in dogs with confirmed acute arterial or venous thrombosis.
- There is insufficient evidence to determine if a specific tPA dosing protocol confers a safety benefit.

### PICO question: Thrombolysis in arterial thrombosis (cats)

In cats with suspected or confirmed arterial thrombosis (P), does use of a thrombolytic agent (I) compared to no thrombolytic agent (C) improve any outcomes (O)?

#### Guidelines

##### 8. Thrombolysis in arterial thrombosis (cats)

- No evidence‐based recommendations can be made regarding the use of systemic or catheter‐directed thrombolytic agents for treatment of acute (<6 h) arterial thromboembolism in cats.
- We suggest that thrombolytic agents can be considered for treatment of acute (<6 h) arterial thromboembolism following an assessment of the risk and benefit in individual patients.

### PICO question: Thrombolytic agents in arterial thrombosis (cats)

In cats with suspected or confirmed arterial thrombosis (P), does use of 1 specific thrombolytic agent (I) compared to any other thrombolytic agent (C) improve any outcomes (O)?

#### Guidelines

##### 9. Thrombolytic agents in arterial thrombosis (cats)

- In cats with confirmed arterial thrombosis, there is insufficient evidence to support the use of one thrombolytic agent over another.
- Of the currently available thrombolytic drugs, tPA has been used most widely for arterial thrombosis in cats, but when indicated the choice of thrombolytic agent will likely be dictated by availability.

### PICO question: Thrombolysis in venous thrombosis (cats)

In cats with suspected or confirmed venous thrombosis (P), does use of a thrombolytic agent (I) compared to no thrombolytic agent (C) improve any outcomes (O)?

#### Guidelines

##### 10. Thrombolysis in venous thrombosis (cats)

- We suggest that in cats with confirmed acute venous thrombosis (<6 h), use of a thrombolytic agent (administered systemically) can be considered when the potential benefits of thrombolysis outweigh the risks of bleeding.

### PICO question: Thrombolytic agents in venous thrombosis (cats)

In cats with suspected or confirmed venous thrombosis (P), does use of 1 specific thrombolytic agent (I) compared to any other thrombolytic agent (C) improve any outcomes (O)?

#### Guidelines

##### 11. Thrombolytic agents in venous thrombosis (cats)

- In cats with confirmed venous thrombosis, there is insufficient evidence to support the use of one thrombolytic agent over another.
- When indicated, the choice of thrombolytic agent will likely be dictated by availability.

### PICO question: Anticoagulants with thrombolysis (cats)

In cats with suspected or confirmed venous or arterial thrombosis (P), does use of a combination of an anticoagulant and a thrombolytic agent (I) compared to use of a thrombolytic agent alone (C) improve any outcomes (O)?

#### Guidelines

##### 12. Anticoagulants with thrombolysis (cats)

- We suggest that combining an anticoagulant with a thrombolytic agent can be considered for treatment of cats with confirmed arterial thrombosis.
- Strong evidence for improved patient‐centered outcomes is lacking and careful consideration of the potential increased risk of bleeding is required.
- No evidence‐based recommendations can be made with respect to the timing of anticoagulant administration in cats undergoing thrombolysis.
- No evidence‐based recommendations can be made with respect to use of anticoagulants in cats undergoing thrombolysis for venous thrombosis.

### PICO question: Antiplatelet agents with thrombolysis (cats)

In cats with suspected or confirmed venous or arterial thrombosis (P), does use of a combination of an antiplatelet agent and a thrombolytic agent (I) compared to use of a thrombolytic agent alone (C) improve any outcomes (O)?

#### Guidelines

##### 13. Antiplatelet agents with thrombolysis (cats)

- We suggest that combining an antiplatelet agent with a thrombolytic agent can be considered for treatment of cats with confirmed arterial thrombosis.
- Strong evidence for improved patient‐centered outcomes is lacking and careful consideration of the potential increased risk of bleeding is required.
- No evidence‐based recommendations can be made with respect to the timing of antiplatelet agent administration in cats undergoing thrombolysis.
- No evidence‐based recommendations can be made with respect to use of antiplatelet agents in cats undergoing thrombolysis for venous thrombosis.

### PICO question: Thrombolytic protocols—Alteplase (cats)

In cats with suspected or confirmed venous or arterial thrombosis (P), does use of a specific protocol (dose, frequency, route) for use of alteplase (I) compared to any other protocol (C) reduce the risk of complications (eg, fatal or nonfatal hemorrhage) or improve any outcomes (O)?

#### Guidelines

##### 14. Thrombolytic protocols—Alteplase (cats)

- No evidence‐based recommendations can be made for a specific protocol for use of alteplase in cats.
