# Supplementary material for: 2022 Update of the Consensus on the Rational Use of Antithrombotics and Thrombolytics in Veterinary Critical Care (CURATIVE) Domain 6: Defining rational use of thrombolytics
Source: J Vet Emerg Crit Care (San Antonio). 2022 Jul 26;32(4):446–70. doi: 10.1111/vec.13227 (PMC9544803; doi:10.1111/vec.13227)
Supplement: Supplementary file 1 — Supporting information [file VEC-32-446-s001.docx]

Supplement 1. Additional literature review for studies neutral to select PICO questions of the 2022 Update of the Consensus on the Rational Use of Antithrombotics and Thrombolytics in Veterinary Critical Care (CURATIVE) Domain 6 - Defining rational use of thrombolytics

**6.7 PICO QUESTION: Thrombolytic protocols – Alteplase (dogs)**

In dogs with suspected or confirmed venous or arterial thrombosis (P), does use of a specific protocol (dose, frequency, route) for use of alteplase (I), compared to any other protocol (C) reduce the risk of complications (eg, fatal or non-fatal hemorrhage) or improve any outcomes? (O)

Four LOE 5 studies^98, 106, 126, 127^ (all case reports or case series and hence considered poor quality) were neutral to the PICO question since they did not compare different rt-PA doses or protocols, but rather just report the clinical use of rt-PA in dogs. One of the included studies includes dog and cat data together, precluding differentiating out dog-specific results.^126^

The first case report described a Yorkshire Terrier with distal aortic thrombosis associated with PLE.^98^ On the day of admission rt-PA was administered at 1 mg/kg as an IV bolus every 60 min for a total of 10 doses, after which pulses improved. By day 6 post-admission the femoral pulses were again undetectable leading to administration of a further 2 doses of rt-PA (1.1 mg/kg IV bolus 60 min apart) that day, and again on day 7 post-admission. Pulse quality was again noted to improve and the dog was discharged on day 9. No adverse effects of rt-PA treatment were noted.^98^

Another case report described a Maltese dog (5.5kg) that developed cranial vena caval thrombus with nearly complete obstruction of blood flow.^106^ Thrombolysis was attempted with 0.4 mg/kg rt-PA IV bolus via a right jugular vein catheter, repeated every 60 minutes, for a total of 4 treatments. Hemorrhage was first noted approximately 30 minutes after the first rt-PA dose at the entry sites of the jugular venous catheter and an esophagostomy tube, and continued throughout the treatment course. Estimated total blood loss was 70-80 mL. A second course of rt-PA was then administered at a lower dose (0.2 mg/kg IV q 60 min for 5 doses), resulting in an additional estimated blood loss of 50 mL. This case report demonstrates that hemorrhage can occur with rt-PA administration in dogs even at relatively low doses (1-2.4 mg/kg cumulative doses administered in a catheter directed fashion).

Finally, Langston et al published a case series and a case report describing alteplase infusions into central venous catheters used for extracorporeal renal replacement therapies.^126, 127^ The retrospective case series describes 17 dogs with catheter dysfunction suspected to be associated with thrombosis.^126^ This case series also included 8 cats, and the dog and cat data is combined. Across all cases alteplase was infused 43 times into 29 catheters (into each lumen), resulting in improved catheter function in 34/43 instances (79%).^126^ The single case report from the same institution describes a dog with a catheter-associated fibrin sheath treated with alteplase instillation in a similar fashion.^127^ In both reports a 2 mg vial of alteplase was routinely diluted with 2 mL of sterile water, to produce a 1 mg/mL solution. This was then infused into the catheter lumen with the goal to fill the lumen (ie, equivalent to the priming volume). For large catheters with a priming volume greater than 2 mL, saline was used to advance the alteplase to the catheter tip. After a median 60 min dwell time the alteplase was then removed from the lumen, but in some circumstances the alteplase was used as a locking solution. Although these reports do not directly address the PICO question they provide some evidence of the way in which alteplase is used in this very specific circumstance.

**6.8 PICO QUESTION: Thrombolysis in arterial thrombosis (Cats)**

In cats with suspected or confirmed arterial thrombosis (P), does use of a thrombolytic agent (I) compared to no thrombolytic agent (C) improve any outcomes? (O)

The majority of the literature (11 published manuscripts^129-139^ and 2 abstracts^140, 141^) were considered neutral to the PICO question; of these only 1 adequately reported the data from a control group (LOE 4, good)^139^, while the remainder lacked control groups, or did not report control group data^132^, and hence were considered to provide low quality of evidence.

The most recent publication on the use of thrombolysis in cats with ATE is also the most robust, a retrospective clinical study using controls, but without randomization (LOE 4).^139^ This study is considered to be of good quality given the multicenter nature and comparison with temporally recent standard of care in a case-control design (1:2 case to control ratio). Cats were eligible for inclusion based on a clinical diagnosis of ATE and involvement of 2 or more affected limbs. Sixteen rt-PA treated cats and 38 control cats were included. Groups were well matched at baseline for proportions of cats with cardiogenic ATE, congestive heart failure, cardiogenic shock, and limb scores. Median time from onset of clinical signs to admission was significantly shorter (3h, range 0-6h) in the rt-PA group, compared to the control group (6h, range 0-48h, P<0.001). rt-PA was administered as either a 1 mg/kg dose IV over 1 h (11 cats), or a 0.1 mg/kg IV loading dose over 1 min followed by 0.9 mg/kg IV over 1-1.5 h (4 cats), or with a progressively increasing infusion rate (17% of a 1 mg/kg dose over 1 min, 46% over 30 min, and 37% over 1 h) in the remaining cat. Mean time from admission to rt-PA administration was 1.5 ± 0.6h. There was no statistically significant difference between groups in the proportion of cats with clinical improvement (9/16 cases [56%], 9/29 controls [31%]), 48 h survival (9/16 cases [56%], 15/38 controls [39%]), or survival to discharge (7/16 cases [44%], 11/38 controls [29%]). The proportions of adverse effects were also no different between groups; specifically, acute kidney injury (3/10 cases [30%], 6/22 controls [27%]), or reperfusion injury (5/10 cases [50%], 11/22 controls [50%]).^139^ However, since this study defined acute kidney injury as a 100% increase in serum creatinine between 2 time points, and reperfusion injury, as an increase in serum potassium concentration above the local laboratory reference interval, the prevalence of these complications could only be assessed in some cats. Six of 16 cats treated with rt-PA did not have follow-up laboratory tests, but 3 of these experienced sudden death on day 2 of hospitalisation.^139^ Overall, this study is considered neutral to the PICO question since it suggests that thrombolysis is not more or less effective at improving outcomes in cats with ATE than no thrombolysis (standard care).

Two experimental models (LOE 3) of aortic thrombosis were considered neutral to the PICO question.^129, 132^ Killingsworth et al conducted a randomized study with controls but its quality was only considered fair given potentially low clinical relevance and numerous limitations of the thrombosis model used.^129^ Adult cats (n=15) were randomized to receive either saline placebo or streptokinase (90,000 IU IV loading dose over 30 minutes, followed by a 45,000 IU/h CRI for 3 h). Although there was evidence of systemic fibrinolysis in the streptokinase-treated group, based on decreased concentrations of fibrinogen, and increased FDPs, PT, thrombin time, and aPTT, they could not differentiate cats in the treatment group from the control group based on aortograms, pelvic limb regional blood flow, or mean clot weight at necropsy.^129^

Hedlund and colleagues published an early experimental study of induced aortic thrombosis in cats that is considered of poor quality, since it was not randomized (although they included a saline control group), the efficacy assessment was not blinded, and efficacy data from the control group was not reported.^132^ Urokinase (at rates ranging from 4,000 U to 200,000 U/h) or saline were infused via an aortic catheter to perform catheter-directed thrombolysis. It seems that thrombolysis did occur in the urokinase treated cats, with maximum thrombolysis of approximately 80%, but in general the results section regarding thrombolytic efficacy is sparse. Since thrombolysis was not compared between the control group and urokinase group, this study did not directly address the PICO question and thus was considered neutral.^132^

The first reports of the use of thrombolytics in cats with naturally developing arterial thrombosis were published by Pion and colleagues, in the late 1980s.^130, 131, 140^ These studies were not peer reviewed and were classified as LOE 5 (case series/reports), poor quality. These sources are included here for completeness however they do not directly address the PICO question. The case series published in abstract form described 6 cats with ATE, 5 bilateral, 1 unilateral, with a mean length of clinical signs of 17 h (range 5-29 h). All were treated with rt-PA at 0.25-1 mg/kg/h as an IV CRI for a total of 1-10 mg/kg. Perfusion was restored in 7/11 affected limbs with a mean time for reperfusion of 5.5 h ± 2h. Adverse effects listed were fever (n=2), bleeding at catheter sites (3), and reperfusion injuries (2). Survival to hospital discharge was 3/6 (50%). Pion also published 2 case reports. The first describes a cat with a 24 h history of posterior paresis and a 12 h history of right thoracic limb paresis, diagnosed with dilated cardiomyopathy and pulmonary edema.^130^ The cat experienced a rapid deterioration and progression to cardiopulmonary arrest after treatment with IV 100 U/kg UFH and rt-PA (1 mg/kg over 1.5 h, then 0.5 mg/kg/h over the next 3 h).^130^ The second case report describes a cat with posterior paralysis, hypertrophic cardiomyopathy and pulmonary edema, diagnosed with ATE via angiography.^131^ Treatment included UFH (5 U/kg IV, followed by 100 U/kg SC q6h), and rt-PA (1mg/kg/h for 1.5h, then 0.5mL/kg/h for 3.5h, concentration not noted). Femoral pulses returned in both legs 3 h after commencing rt-PA and the cat was discharged 48 h after admission with only mild unilateral conscious proprioception deficits. Nonetheless euthanasia resulted 18 days later after recurrence of posterior paresis.^131^

Additional case reports detailing thrombolysis in cats with ATE have also been published (all LOE 5, poor).^125, 133, 135^ Koyama et al described a cat with bilateral distal ATE and HCM treated with urokinase (12,000 U IV over 30 min, then daily on day 1 and 2), warfarin (0.05 mg/kg PO q12h), dipyridamole (12.5mg PO q12h), and UFH (100 IU/kg SC q12h). Clinical signs remained unchanged on day 3, prompting local intra-arterial administration of urokinase (total dose 174,000 U) until patency of the right and left femoral arteries was restored. By day 5 the cat regained the ability to ambulate and was discharged with a near normal gait on day 9.^133^ Adverse effects were not described. Huang et al described a cat that presented with pelvic limb paresis of unknown duration diagnosed with distal ATE.^134^ Streptokinase was administered at 90,000 IU IV as a CRI over 20 min, followed by 45,000 IU as an IV CRI q8h for 2 additional doses, after which time the cat had regained motor function in both pelvic limbs. The only reported adverse effects were a mild transient azotemia.^134^ Another more recent case report also documents the successful medical thrombolysis of a distal ATE with IV injection of rt-PA (alteplase) in addition to treatment with heparin and an antiplatelet agent. The cat had return of motor function within 12 h, return to ambulation with residual paresis in 48 h, and ultrasonographic resolution of thrombosis, without significant adverse effects.^135^

Moore et al (LOE5, Fair) published a historical case series of 46 cats with suspected arterial thrombosis treated with streptokinase.^136^ The majority (41) had bilateral hindlimbs affected, while lesser numbers has a single pelvic limb (4), or thoracic limb (1) affected. Varying streptokinase dosing protocols were reported with a median dose of 47,345 U/kg (range 18,857-158,529 U/kg), and a median duration of infusion of 4 h (1-28h). The most common protocol (n=25) was 90,000 U then 45,000 U/kg for 3 h, with the duration of infusion extended to 4 h (1 cat), 5 h (3 cats), 6 h (1 cat), or 7 h (1). A single cat received only the 90,000 U bolus, and a single cat was administered a 250,000 U bolus over 1 h. Regarding thrombolytic efficacy, 25 (54%) regained arterial pulses within 2-24h of streptokinase administration, 14 (30%) regained motor function, and 15 (33%) survived to hospital discharge. Of those cats that regained motor function, 11 were within 9-24h of streptokinase while the remaining 3 cats had delayed restoration of motor function after leaving the hospital. Survival to discharge was not associated with any features of streptokinase dosing, including time to administration. There was no difference in the median time from onset of clinical signs to commencement of streptokinase treatment between survivors (6h, range 2-12h), and non-survivors (5h, range 1-20, P = 0.356). Adverse effects were report commonly including increased dyspnea (n=14, 30%), confirmed hyperkalemia (14, 30%), and clinical evidence of bleeding (18, 39%). Common clinical manifestations of hemorrhage included hematuria, bleeding from catheter sites, and oral or rectal mucosal bleeding. Three cats required blood transfusions for severe bleeding, although overall the occurrence of bleeding did not affect survival.^136^

Whelan et al published a historical case series in abstract form (LOE 5, poor) that is considered neutral to the PICO question.^141^ Twelve cats with cardiogenic ATE were treated with urokinase (dose and time from onset to treatment not reported). Efficacy is difficult to discern from the reported statistics on return or improvement of pulses, and motor function, but survival to discharge was 5/12 (42%), while 7/12 were euthanized. No clinical bleeding was reported, but 3 cats were reported to develop reperfusion injuries.^141^

Welch et al conducted a single center prospective study to evaluate the clinical response and side effects of 2 dosing protocols of rt-PA in cats with ATE that also considered neutral to the PICO question (LOE5, fair).^137^ Eleven cats with a median duration of clinical signs of 4 h prior to presentation (range 2-12 h) were enrolled. Cats were randomized to receive rt-PA within 1 h of presentation. Group A cats received 5 mg/cat of tPA as an IV CRI over 4 h, while group B had accelerated dosing (same total dose of 5 mg but over 1.5 h). Specifically, the accelerated dosing protocol was an initial 1 mg IV bolus, followed by 2.5 mg IV over 30 min, and the remaining 1.5 mg IV over 1 h. In this study, antiplatelet and anticoagulant drugs were not permitted for the first 24 h. Two cats in each group received an additional dose of tPA (5 mg IV over 4 h) after 1 or more limbs continued to have no pulse or motor function. Although 53% had restoration of pulses and 33% had restoration of motor function within 24 h, only 3/11 (27%) survived to discharge. Adverse effects were reported in all cats, including azotemia (n=5), neurologic signs (5), cardiac arrhythmias (5), hyperkalemia (4), acidosis (2), and sudden death (1). The high frequency of adverse events led the investigators to terminate the study early, and this resulted in the inability to compare dosing protocols as planned.^137^

Finally, a study by Oh and colleagues included both dogs and cats without clear differentiation of which results were in cats versus dogs.^138^ Dogs and cats with aortic thrombotic disease were randomly selected to receive either an IV CRI of rt-PA (Group A; 0.5-1 mg/kg/h with a total dose of 20 mg over more than 4 h) or an accelerated dosing protocol (Group B; 1-5 mg/kg IV bolus rt-PA followed by a 0.5 mg/kg/h CRI for a total of 20 mg). Group A and B were further subdivided into low and high dose groups, although the presentation of the figure in the report makes this differentiation unclear. Inclusion criteria included a diagnosis of ATE based on clinical signs (some also had diagnostic imaging) with a duration of < 48 h. Outcome was analyzed from 17 patients, of which it appears 13 were cats. Overall, 8/17 (47%) survived to discharge, but only 6 of these 8 patients had improvement in their limb score at 48h. Eight animals in total had adverse events reported, 4 of which were non-survivors. Adverse events included mild azotemia (3), reperfusion injury (2) progressing to multiple organ dysfunction in one, mucosal bleeding (2), and seizures (2). Although the authors draw conclusions regarding the efficacy of different dosing regimens,^138^ samples sizes are too small, and data reporting appears inaccurate or inconsistent, such that these conclusions are inadequately founded. As such, this study remains neutral to the PICO question.

**6. 12 PICO QUESTION: Anticoagulants with thrombolysis (Cats)**

In cats with suspected or confirmed venous or arterial thrombosis (P), does use of a combination of an anticoagulant and a thrombolytic agent (I), compared to use of a thrombolytic agent alone (C) improve any outcomes? (O)

Eight reports were neutral to the PICO question (1 LOE 4 poor,^139^ 7 LOE 5 fair-poor studies^130, 131, 133-136, 143^). All identified studies addressed arterial thrombosis, rather than venous thrombosis. The neutral studies are only considered of only fair or poor quality, because they did not directly address the PICO question.

Moore et al., (LOE5, fair) published a case series of 46 cats with suspected arterial thrombosis affecting at least 1 limb describing improved survival in those given streptokinase and UFH versus streptokinase alone (P=0.052).^136^ The study was highly relevant to the PICO question but the statistical methods and some relevant results are incompletely described. Streptokinase dosing was not standardized but rather was determined by the clinician and varied widely (bolus of 20,000-25,0000 units, followed by a CRI over 1-28h at varying doses). The median duration of streptokinase administration was 4h, with a median dose of 47,345 U/kg (min-max 18,857-158,529 U/kg). Unfractionated heparin was administered to some cats, although the number is not stated, at doses from 50-232 U/kg q6h SC. The timing of streptokinase nor UFH relative to the onset of clinical signs of thrombosis were not described. Overall, 15/46 cats survived to hospital discharge, cats treated concurrently with UFH were deemed more likely to survive than those not treated with UFH (P=0.052, which is above the study’s *a priori* threshold).^136^ Additionally, anticoagulants were continued at home in 14/15 cats that survived to discharge. Specifically, 12 received coumadin, of which 2 were euthanized after coumadin related hemorrhagic complications, and 2 received dalteparin. It is unclear how many cats received UFH, and when coumadin or dalteparin were commenced relative to streptokinase or UFH administration.^136^ Of note, the CURATIVE guidelines recommend that other anticoagulants (UFH, LMWH or direct Xa inhibitors) are used in preference to warfarin / coumadin in cats with thrombosis.^5^

One case controlled clinical study (LOE 4, poor)^139^ was considered neutral to the PICO question and of poor quality relative to guideline 6.12, since it was not designed to answer this PICO question and the majority of cats received an anticoagulant. In the rt-PA group (n=16), 6 cats also received enoxaparin, while 5 cats also received UFH. Similarly in the standard care control group that did not receive rt-PA (n=38), 13 cats received UFH, 9 enoxaparin, and 8 nadroparin. There was no statistically significant difference between the rt-PA and control groups in the proportion of cats experiencing clinical improvement or surviving to 48 h or to discharge. Additionally, there was no difference in the rate of adverse events (acute kidney injury, or reperfusion injury) between groups.^139^

A small number of case reports (LOE 5)^130, 131, 133-135^ describe cats treated with concurrent thrombolysis and anticoagulation, with mixed outcomes. Given differences in treatment protocols, it is difficult to make any inferences about the benefits or risks of the addition of an anticoagulant to thrombolysis.

Four case reports (LOE5, poor)^130, 131, 133, 135^ specifically describe the use of UFH in combination with thrombolysis in cats with ATE and are deemed neutral to the PICO question. The first case received a combination of IV rt-PA thrombolysis and UFH (100 U/kg SC).^130^ The cat developed severe hyperkalemia and cardiac arrest within 2 h of treatment, and ultimately died.^130^ The second case report described a cat with ATE that was treated with a combination of rt-PA and UFH (5 U/kg IV once then 100 U/kg SC q6h while hospitalized), in addition to aspirin (25 mg/kg PO q72h), and had successful reperfusion in hospital, with return of ambulation.^131^ However, the cat developed rethrombosis, suspected based on recurrent paraparesis, 18 days later and was euthanized.^131^ Koyama et al describe a cat treated with urokinase for a distal ATE that also received UFH and warfarin.^133^ Urokinase was administered systemically on the first 2 days of hospitalisation, followed by catheter-directed intra-aortic administration on the third day. Daily IV administration of urokinase then continued until day 9. Concurrently, the cat also received UFH 100 IU/kg SC q12h, and warfarin 0.05 mg/kg PO q12h, that were commenced on the day of admission and continued throughout hospitalisation, in addition to an antiplatelet agent. The cat regained the ability to walk on day 5 of hospitalization, was discharged on day 9, and was reported to be doing well on day 11 (the last day of follow-up). Complications, such as hemorrhage or reperfusion injury, were not reported.^133^ The final case report of the successful treatment of a cat with ATE could only be reviewed in abstract form, but documented the use of heparin in combination with rt-PA, and an antiplatelet agent.^135^

Two studies included cats that received both a thrombolytic agent and LMWH, specifically dalteparin.^134, 143^ Saida et al explored the use of dalteparin alone compared to dalteparin and rt-PA in a historical case series of cats with ATE, but do not have a rt-PA only group, and thus this study does not directly address the PICO question (LOE 5, poor).^143^ Eight cats (Group A) received dalteparin alone, at dose of 50-200 U/kg IV or SC, q12-24h for 1-17 days. Seven cats (Group B) received a combination of dalteparin (100-200 U/kg IV or SC, q12h for 2-13 days) and monteplase (27,500-72,000 U/kg IV on day 1). Additionally, a single case report describes the concurrent use of dalteparin (100 U/kg, route not reported) with streptokinase, although a frequency of dalteparin administration is not listed in the report, thus it is unclear if only a single dose was given or if the drug was continued.^134^ Thrombolysis with return of function occurred, and no bleeding complications were noted in this case.^134^
